# Ecological inferences on invasive carp survival using hydrodynamics and egg drift models

**DOI:** 10.1038/s41598-024-60189-1

**Published:** 2024-04-25

**Authors:** Ruichen Xu, Duane C. Chapman, Caroline M. Elliott, Bruce C. Call, Robert B. Jacobson, Binbin Wang

**Affiliations:** 1 Department of Civil and Environmental Engineering, University of Missouri, Columbia, MO 65211 USA; 2 U.S. Geological Survey Columbia Environmental Research Center, Columbia, MO 65211 USA; 3 School of Natural Resources, University of Missouri, Columbia, MO 65211 USA; 4 Missouri Water Center, Columbia, MO 65211 USA

**Keywords:** Ecological modelling, Invasive species, Riparian ecology, Ecology, Ecological modelling, Freshwater ecology, Invasive species, Riparian ecology

## Abstract

Bighead carp (*Hypophthalmichthys nobilis*), silver carp (*H. molitrix*), black carp (*Mylopharyngodon piceus*), and grass carp (*Ctenopharyngodon idella*), are invasive species in North America. However, they hold significant economic importance as food sources in China. The drifting stage of carp eggs has received great attention because egg survival rate is strongly affected by river hydrodynamics. In this study, we explored egg-drift dynamics using computational fluid dynamics (CFD) models to infer potential egg settling zones based on mechanistic criteria from simulated turbulence in the Lower Missouri River. Using an 8-km reach, we simulated flow characteristics with four different discharges, representing 45–3% daily flow exceedance. The CFD results elucidate the highly heterogeneous spatial distribution of flow velocity, flow depth, turbulence kinetic energy (TKE), and the dissipation rate of TKE. The river hydrodynamics were used to determine potential egg settling zones using criteria based on shear velocity, vertical turbulence intensity, and Rouse number. Importantly, we examined the difference between hydrodynamic-inferred settling zones and settling zones predicted using an egg-drift transport model. The results indicate that hydrodynamic inference is useful in determining the ‘potential’ of egg settling, however, egg drifting paths should be taken into account to improve prediction. Our simulation results also indicate that the river turbulence does not surpass the laboratory-identified threshold to pose a threat to carp eggs.

## Introduction

Bighead carp (*Hypophthalmichthys nobilis*), silver carp (*H. molitrix*), black carp (*Mylopharyngodon piceus*), and grass carp (*Ctenopharyngodon idella*), are considered invasive in North America. These species were imported into North America in the 1970’s to support aquaculture and escaped into the wild where they alter aquatic environments and food webs, resulting in undesirable ecological consequences^1–3^. On the other hand, these carp species are important food sources in China, yet their populations in their native environment have been declining due to over-fishing and the negative effects on fish habitats resulting from dam construction^4,5^. As either native or invasive species, it is of great importance to understand their life cycles in order to identify potential intervention strategies to control their populations^6^.

These rheophilic, broadcast-spawning carps exhibit prolific reproduction, with a single female carp capable of producing between 100,000 and one million eggs annually^7^. Carps typically engage in spawning during the spring and summer months when the temperature is within a range favorable for successful reproduction (peaking at roughly 20–24 ^∘^C) and during periods of high flows^8,9^. They select specific locations for spawning characterized by high turbulence, including rocky rapids, riffles, islands, river confluences, and bends. This choice helps prevent the settling of eggs onto the riverbed, as sediment burial causes high mortality^10^. Within 3–5 h after spawning, eggs absorb a large amount of water in a process known as water hardening, leading to an increase in egg size and decrease in egg density. The water-hardening process leads to a decrease in settling velocity by approximately 70%, making eggs more likely to suspension in the water column^10,11^.

After spawning and fertilization, the drift stage of carp eggs begins, a critical early-life stage in carp recruitment. Eggs hatch in approximately 30 h at optimal temperatures^10,12^. During the drift stage before hatching, eggs are susceptible to predation, relying entirely on river currents and turbulence to remain suspended until hatch. After hatching, larval carp remain in the drift for a period, but they can behaviorally avoid settling^10,12^. Because hydrodynamics plays a critical role in the suspension, dispersion, and transport of carp eggs across various scales in rivers, numerous studies have been conducted to explore river hydraulics and turbulence in relation to suitable carp spawning grounds, survival potential, and hatch locations^13–16^. A key survival condition is the necessity for eggs to remain suspended in the water column throughout the entire egg drift stage, or at the very least, to avoid settling and being buried by sediment. Consequently, assessing whether river hydrodynamics can support this condition is a fundamental step in gauging recruitment success.

Flow velocity has been used as a simple indicator for assessing the suspension of eggs in rivers. For instance, Kocovsky et al.^17^ used a threshold velocity of 0.7 m/s as suitable for the spawn-to-hatch environment. Selection of 0.7 m/s is based on early literature with limited mechanistic studies^9,18^. Lower critical flow velocities were also reported in the literature. Tang et al.^19^ suggested a value of 0.25 m/s based on a flume experiment, which agreed with some early field observations in the Yangtze River. Murphy and Jackson^20^ found that mean velocities of 0.15–0.25 m/s allowed for egg suspension in four tributary rivers of the Great Lakes. Guo et al.^21^ suggested a critical flow velocity of 0.3 m/s in a flume experiment. Because rivers are largely non-uniform and vary in size and morphology, selecting a specific flow velocity as the sole empirical indicator for assessing suitability of carp recruitment is rather challenging.

While using flow velocity as an indicator for examining egg suspension or settling might be practical, it does not fully represent the underlying physics, especially in areas where turbulence is not well correlated with mean flow velocity. To account for the mechanism of egg suspension, Garcia et al.^22^ proposed three different criteria involving the ratio of shear velocity and egg settling velocity, the ratio of vertical turbulence intensity and egg settling velocity, and the Rouse number to predict the suspension and settling of carp eggs. In their laboratory experiment, they observed that 65% of eggs remained in suspension with a mean flow velocity of 0.07 m/s, corresponding to a Rouse number of 1.32 and shear velocity of 0.004 m/s. At higher flow velocities of 0.2 and 0.4 m/s, with Rouse numbers of 0.57 and 0.58 and shear velocity of 0.008 and 0.016 m/s, respectively, all eggs were in suspension. These observations agree well with the empirical values of Rouse number classification for sediment transport for bedload, partial suspension, full suspension, and washload^23^. Therefore, using these parameters is better supported by the mechanism of particle suspension compared to velocity alone.

Given the above simple criteria of using shear velocity or Rouse number, hydraulic models or measurements can be used to infer whether a stream or a river reach can support a favorable environment for egg suspension in the egg-drift stage^17^. In addition, three dimensional hydrodynamic models can provide additional insights into the spatial distributions of potential egg settling zones, given the strong spatial heterogeneity of river turbulence^24–26^. In this paper, we use an 8-km reach in the Lower Missouri River as representative of channelized segments of the Upper Mississippi River basin where carps are established. We used computational fluid dynamics (CFD) modeling to explore the overall suitability for egg drift and to infer potential egg settling zones, with an emphasis on understanding the spatial distributions of hydrodynamics associated with in-stream hydraulic structures, river morphology, and strong topographic gradients on the riverbed. Specifically, we examine the criteria of egg suspension and evaluate the locations where the hydrodynamics are unfavorable for suspending eggs. Our objective is to evaluate whether the potential egg settling zones based on hydrodynamic inference would agree with entrapment locations that can be estimated using drift models. We additionally evaluate whether turbulence conditions indicated in the model approach criteria for turbulence-induced damage to carp eggs as determined in laboratory studies.

## Methods

### Study site

**Figure 1:**
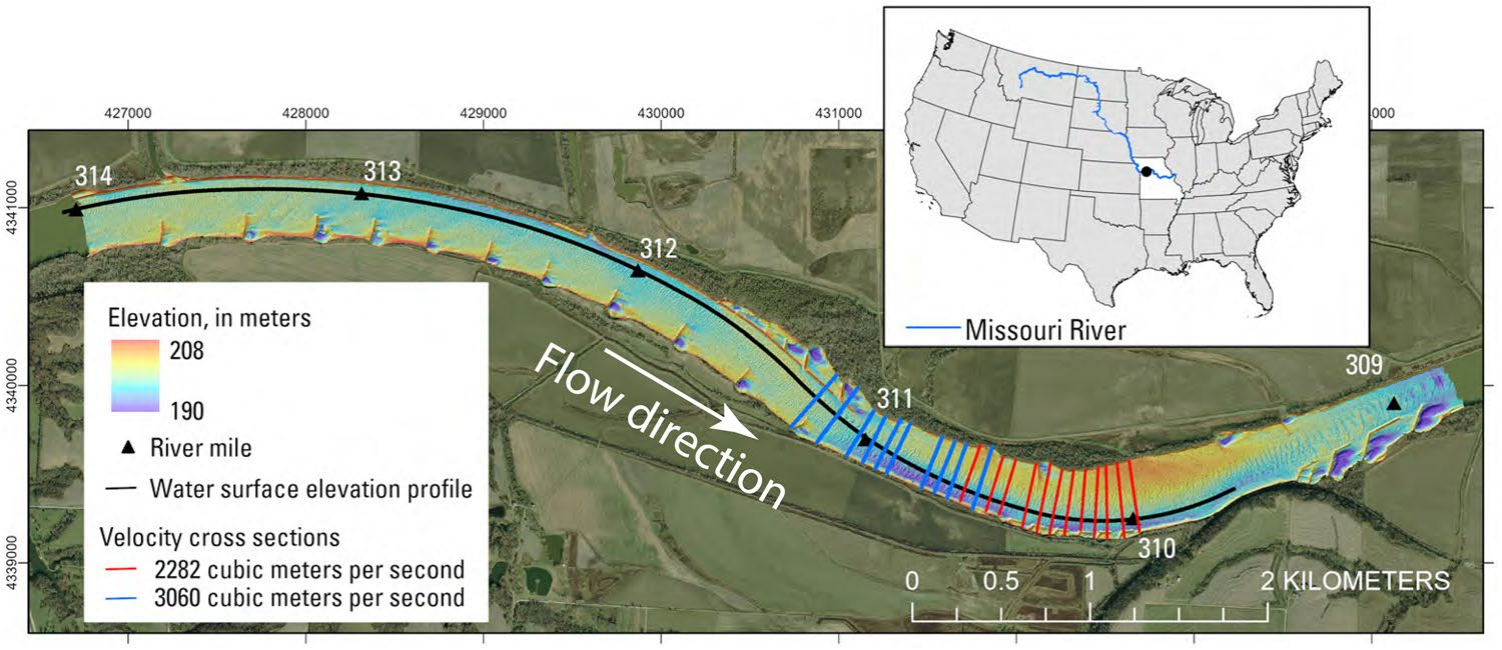
Bathymetry map of the study site in the Lower Missouri River. Black line represents the measurement of water surface elevation. Black triangles represent the river miles measured from the confluence with the Mississippi River near St. Louis, Missouri. Twelve red lines represent the cross sectional transects of velocity measurement at $Q = 2282$ m^3^/s. Ten blue lines represent the cross sectional transects of velocity measurement at $Q = 3060$ m^3^/s. Map was generated with ArcGIS Pro v. 3.2 https://www.esri.com/en-us/home. Basemap is U.S. Army Corps of Engineers Imagery, 2012. River miles are from the U.S. Army Corps of Engineers, 1960, https://www.nwk.usace.army.mil/Missions/Civil-Works/Navigation/.

The study site is a selected reach in the Lower Missouri River near Lexington, Missouri (Fig. 1). The reach is approximately 8 km long with a sinuosity index of 1.12. The mean bankful width is 331.4 m. The bed is mostly covered by medium and coarse sand ($D_{50}$ = 0.55 mm) with fine muddy materials (< 0.125 mm) near the banks and close to the dike fields^27,28^. The mean annual discharge is approximately 1700 m^3^/s measured at a U.S. Geological Survey (USGS) gaging station approximately 24 km downstream (station no. 06895500, Waverly, Missouri, USGS). The reach is representative of rivers that have been highly engineered to support navigation and bank stability, with complex hydraulic conditions where water flows around and over the rock channel-training structures^29,30^. This reach has been used as the main site for model development stage of SDrift^31,32^, an egg drift model used in this study. The previous studies have accumulated substantial data for the bathymetric-topographic digital elevation model (DEM), water surface elevations, and cross-channel velocity profiles^33^, which have been used for calibration and validation of our CFD model.

### Hydrodynamic model

The flow was simulated using FLOW-3D HYDRO with a Reynolds-averaged Navier-Stokes (RANS) solver and a Re-Normalization Group (RNG) modified $k-\varepsilon$ turbulence sub-model. The model was set up for solving the steady-state flows under four discharge conditions ( *Q* = 1342, 2282, 3060 and 4219 m^3^/s, referred to as $Q_1$ to $Q_4$ conditions), which correspond to approximately 45–3% daily flow exceedance during spawning season. A Cartesian mesh with a final size of $4 \times 4 \times 0.4$ m in the east-north-up coordinate system was used after a mesh independence study to evaluate optimal mesh dimensions^31^.

The upstream and downstream boundary conditions were set to the measured flow discharge and calculated hydrostatic pressure from the measured water-surface elevation, respectively. The model was calibrated by adjusting the roughness coefficient until the simulated water-surface elevations agree with the measured data, where the water-surface elevations were measured using a ship-mounted, real-time corrected kinematic global navigation satellite system (RTK-GNSS). The measured cross-channel velocities at 22 locations at two flow conditions ($Q = 2282$ and 3060 m^3^/s) were used to evaluate model performance, where the velocities were measured using a ship-board acoustic Doppler current profiler (ADCP, Workhorse Rio Grande, Teledyne, Inc) at each cross section with four repeated transects. The ADCP had a vertical resolution of 0.5 m and horizontal resolution of 1 m. The velocities within 1 m below the water surface and within 1 m above the river bed were not measured due to instrument blanking distance and measurement noise. Additional details on model calibration and evaluation are in Li et al.^31^.

### Egg drift model

The egg drift model SDrift was used for egg transport modeling in this study^31^. This model uses Lagrangian particle tracking to simulate the transport of carp eggs, where turbulent fluctuations are modeled using an explicit solution for the Langevin equation, i.e., the Markov-chain continuous random walk (CRW) algorithm^34–36^. The density and diameter of carp eggs were determined as a function of post-fertilization time and water temperature based on the regression equation to the laboratory measured data^11^. The details of regression can be found in^31^. The time-varying characteristics of eggs result in evolving egg settling velocity in the water, which is determined based on the drag law for spherical particles^37^.

SDrift was incorporated with the CFD model outputs to predict transport of silver carp eggs in the selected reach. A broad surface-spawning event across the entire cross section at an upstream location in the model ($x =$ 427,130 m, near River Mile 314) was simulated by releasing 6600 model eggs on the water surface at 33 locations^31^. All eggs were tracked until they were transported outside the downstream boundary or ‘entrapped’ in the model domain determined by the model criterion.

### Criterion of egg entrapment from the egg drift model

SDrift allows the simulated eggs to be ‘entrapped’ if they are stationary for a pre-defined duration. The entrapment would occur if a simulated egg is transported into a low velocity zone and eventually loses its momentum. From the model evaluation, entrapment primarily occurs in the region with high topographic gradients, e.g., near the bank and hydraulic structures. A duration of 30 s was used here to determine the entrapment, i.e., if a simulated egg does not move for 30 s, it would be considered entrapped and would no longer be tracked. Although the entrapment does not necessarily provide a certain prediction of egg settling, it offers insight into locations where the eggs may be stopped and eventually buried by bed sediment. The selection of a 30-s duration is somewhat arbitrary. From a physics standpoint, this duration should ideally exceed the largest turbulent time scale. However, due to the extensive spatial scale of the modeled reach and the river-training structures, the turbulent time scale varies significantly across space. Furthermore, both the spatial resolution in the CFD simulation and the temporal resolution in particle tracking have the potential to influence particle movements and their entrapment. Therefore, determining the optimal duration requires further investigation in future studies.

### Criterion of egg suspension and settling from the hydrodynamic model

Suspension of carp eggs depends on whether the flow can provide adequate upward motions that overcome their settling. Analogous to sediment suspension and transport^38^, several means have been used to quantify the settling and suspension of carp eggs in turbulent flows. Here we analyze three parameters following Garcia et al.^22^: the ratio between shear velocity and settling velocity, the ratio between vertical turbulence intensity and settling velocity, and the Rouse number.

#### Shear velocity

Shear velocity ($u_*$) is a velocity scale defined from the bed shear stress. The ratio of shear velocity and particle terminal velocity ($w_t$), a so-called movability number ($M_* = u_*/w_t$), has been used to classify sediment transport^39^. Different critical values have been proposed to define particle suspension^38,39^. Here, the critical value of 1.0 is used following the studies of carp eggs^20,22^: locations with $u_*/w_t < 1$ are the potential settling zones of carp eggs, where particle terminal velocity is the egg settling velocity ($w_t=V_{\text {egg}}$).

Because shear velocity only represents the bed shear but does not provide the vertical variability in the water column, we applied a scaling method so that potential egg suspension and settling can be evaluated in the entire water column. Using the relationship between bed shear and turbulence kinetic energy (TKE)^40,41^, i.e., $\tau _b = C_1 \rho k$ with $C_1=0.19$^40^, the movability number can be estimated at every grid point using the TKE determined from the CFD simulation:

1 $$\begin{aligned} M_* = \frac{\sqrt{C_1 \text {TKE}}}{V_\text {egg}} = \frac{0.44 \sqrt{\text {TKE}}}{V_\text {egg}} \end{aligned}$$

The potential egg settling zones were then determined based on $M_*<1$.

#### Vertical turbulence intensity

The vertical turbulence intensity ($w^{\prime }_\text {rms}$) is a direct parameter to quantify the turbulent velocity scale in the vertical direction, which can be used to define the initiation of particle suspension^38^. Therefore, we also calculated the ratio between $w^{\prime }_\text {rms}$ and $V_\text {{egg}}$ as the second indicator for egg settling: locations with $w^{\prime}_{rms}/V_{egg}<1$ are the potential settling zones of carp eggs. Here, we estimated $w^{\prime }$ based on anisotropy of turbulent fluctuations in open channel flows:

2 $$\begin{aligned} w^{\prime }_\text {rms} = \sqrt{\frac{2D_w^2 \text {TKE}}{D_u^2+D_v^2+D_w^2}} \end{aligned}$$

with $D_u = 2.30$, $D_v = 1.27$, and $D_w = 1.63$^42^. This gives $w^{\prime }_\text {rms}/V_\text {egg} = 0.75 \sqrt{\text {TKE}}/V_\text {egg}$ where TKE was obtained from the CFD simulations.

#### Rouse number

In sediment transport, the Rouse number has been used to describe the suspended load^38^. The Rouse number is defined as $Ro = w_t/(\beta \kappa u_*)$ with $w_t=V_\text {egg}$ for carp eggs, where $\kappa$ is von Kárman constant and $\beta$ is a coefficient related to diffusion of particles^22,23^:

3 $$\begin{aligned} \beta = \min \left[ 1+2 \left( \frac{w_t}{u_*} \right) ^2, 3 \right] \end{aligned}$$

The Rouse number (*Ro*, also used as *Z* or *P* in the literature), can be used to classify the sediment transport similar to the movability number. Hearn^23^ suggested that sediment particles are in 100% suspension or wash load when $Ro < 1.2$; particles are partially suspended when $1.2< Ro <2.5$; particles are predominantly transported by bedload if $Ro>2.5$. Here, we use 1.2 as the criterion, such that the potential egg settling zones were determined based on $Ro > 1.2$.

## Results and discussion

### Model calibration and evaluation

**Figure 2:**
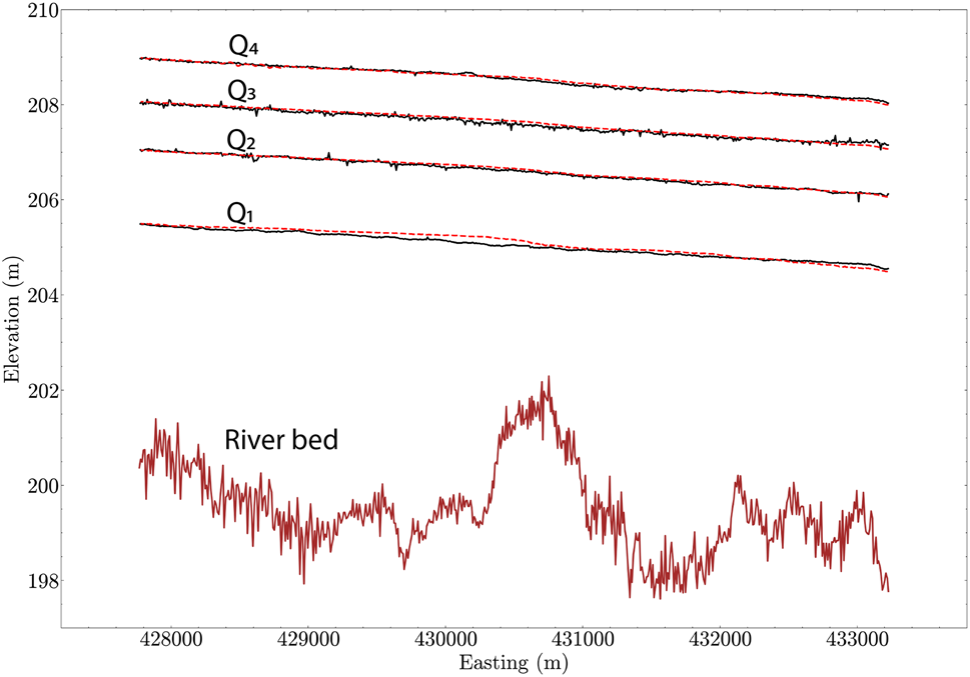
Result of model calibration using the measured water surface elevation for four discharge conditions from Li et al.^31^ and Elliott et al.^33^. Black solid lines are measured data. Red dashed lines are modeled results.

The model calibration results for water-surface elevation are shown in Fig. 2 for four flow conditions^31^. The elevation of river bed in the main channel is also plotted for reference. The root-mean-square-error (RMSE) in the water surface elevation between the measurement and modeling is 0.07, 0.03, 0.04, and 0.03 m, for $Q_1$ to $Q_4$, respectively. The RMSE is considered to be small compared to the length of the reach and the water depths.

**Figure 3:**
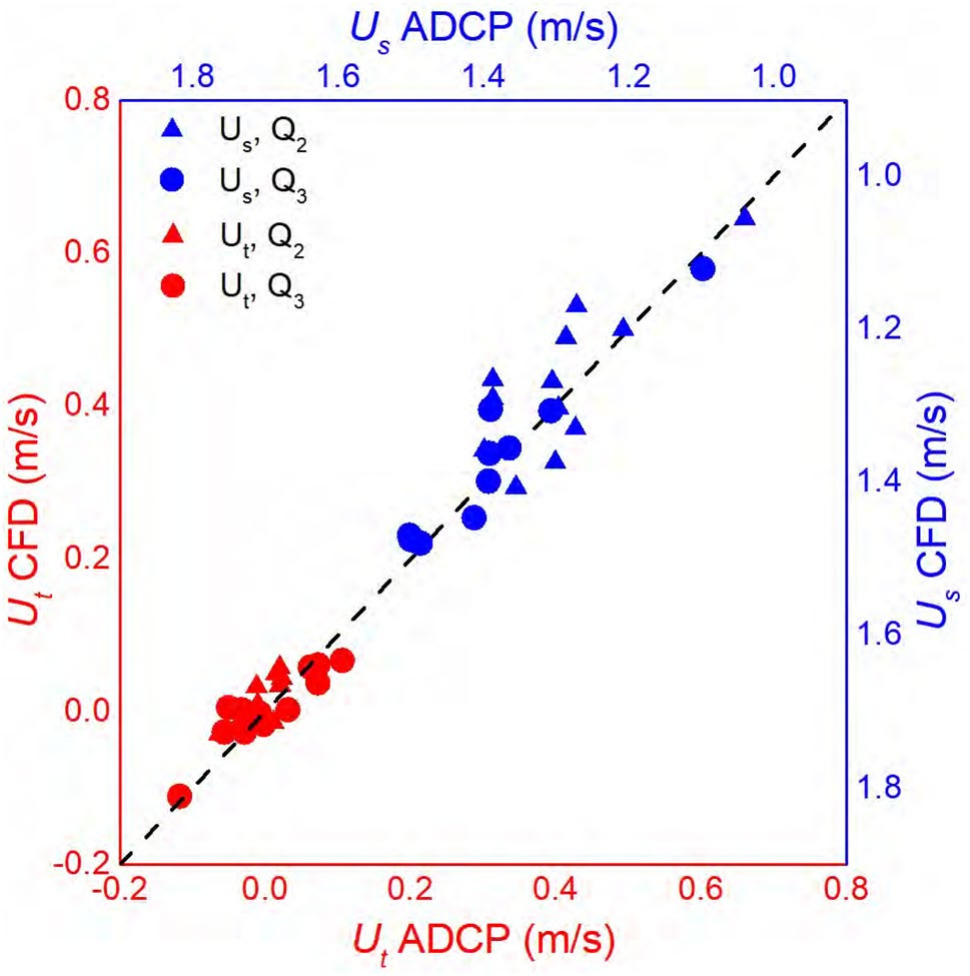
Comparison between computational fluid dynamics (CFD) modeled and acoustic Doppler current profiler (ADCP) measured velocities in the streamwise direction ($U_s$) and transverse direction ($U_t$) at 22 cross sections under the two surveyed conditions $Q_2$ and $Q_3$^33^. The 1:1 dashed line represents perfect agreement.

The measurement-modeling comparison of double-averaged velocities over the flow depth and the cross section in both streamwise ($U_s$) and transverse ($U_t$) directions is given in Fig. 3 for two measured conditions ($Q_2$ and $Q_3$). The RMSE of $U_s$ and $U_t$ is 0.055 and 0.028 m/s, much smaller than the mean flow of 1.29 and 1.38 m/s in the measured cross sections for $Q_2$ and $Q_3$, respectively. The direct measurement-modeling comparison in all 22 cross sections is given in the supplementary file (Figs. S1 and S2).

### Mean flow characteristics

**Figure 4:**
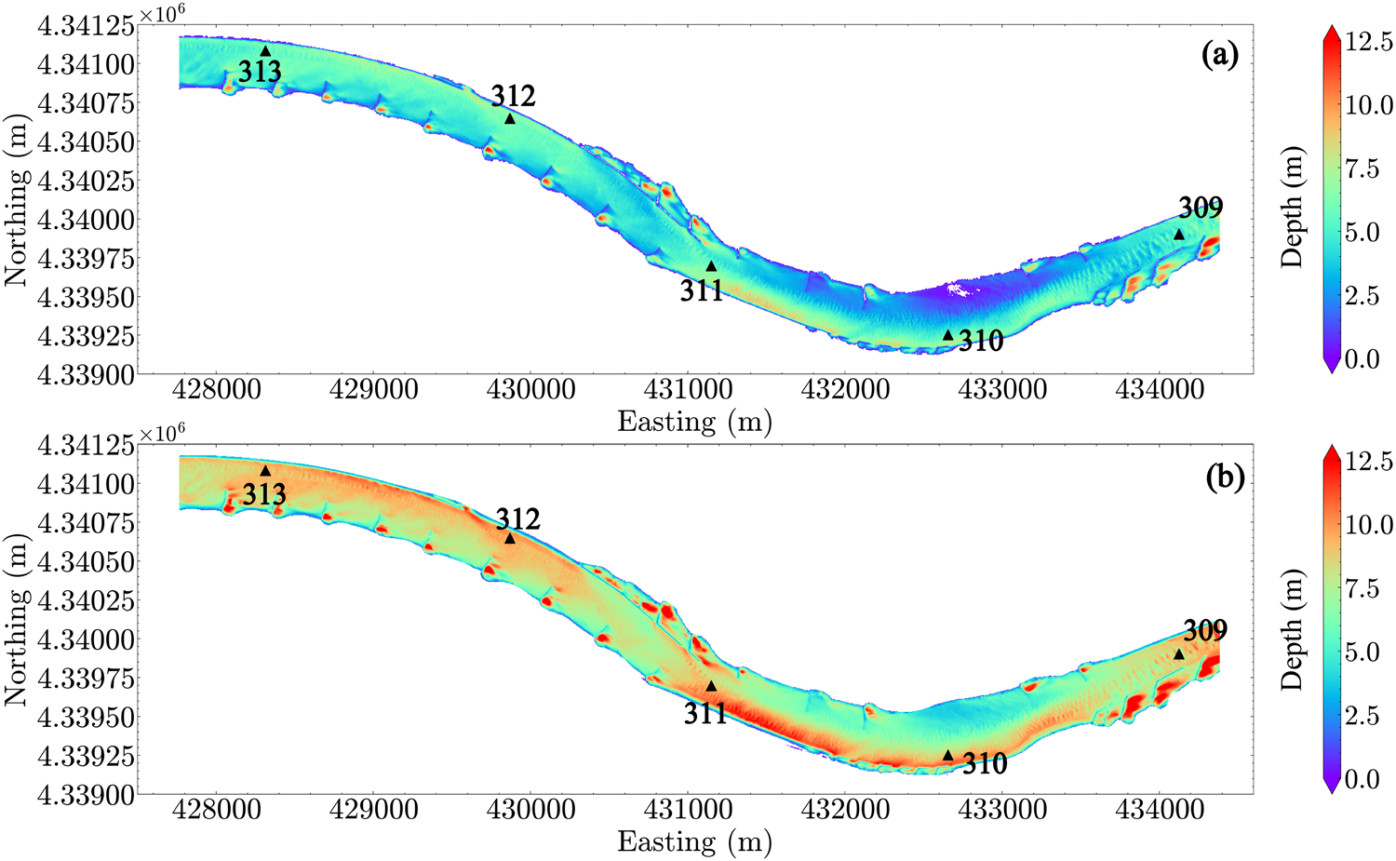
Flow depth in the reach: (**a**) $Q_1$; (**b**) $Q_4$. River miles 309–313 are indicated in the plot by black triangles.

**Figure 5:**
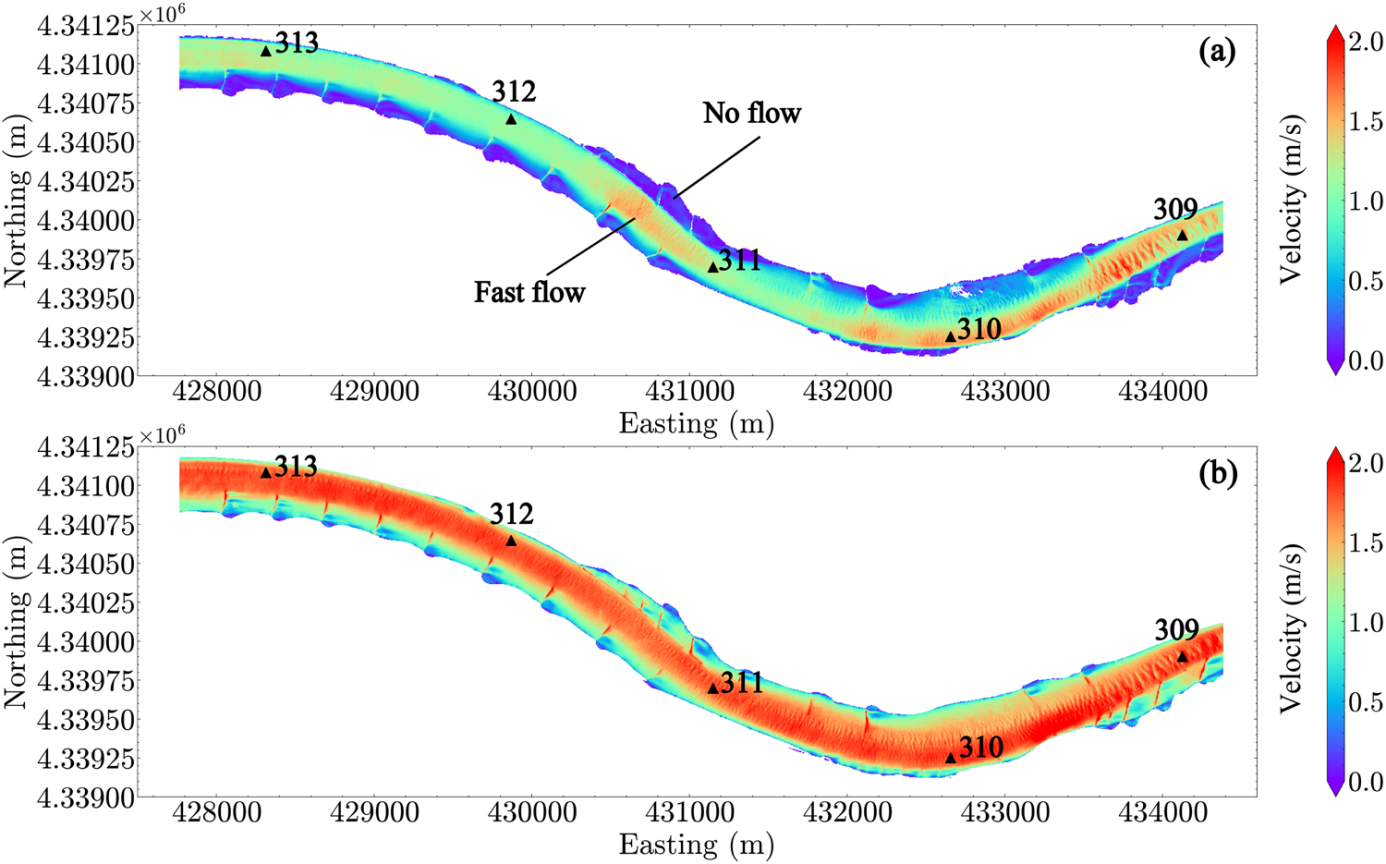
Depth-averaged flow velocity in the reach: (**a**) $Q_1$; (**b**) $Q_4$. River miles 309–313 are indicated in the plot by black triangles.

The CFD simulated flow depth and depth-averaged flow velocity for two out of four conditions are shown in Figures 4 and 5. Greater depths are located downstream from the dikes (i.e., in scour holes) and near the right bank at the upstream bend (i.e., Easting 431,000–432,000 m, downstream of river mile 311). Shallower depths are located upstream from the dikes and along the left bank in the downstream bend (i.e., Easting 432,000–433,500 m, in the vicinity of river mile 310).

Flow velocities are greater at a higher discharge, and are strongly related to the in-stream hydraulic structures: high velocities are located within the main channel and low velocities are located close to the dike areas and both sides of the bank. For $Q_1$, the L-head dikes on the left bank around Easting 430,500–431,000 m (upstream of river mile 311) block the flow into the left bank, resulting in channel narrowing and an area of localized higher velocity. Relatively faster velocities are also located close to the right bank from Easting 432,000–433,500 m (in the vicinity of river mile 310) and then shaped by the L-head dike at Easting 433,500–434,500 m (between river miles 309 and 310). When water enters the L-head dike area at Easting 430,500–431,000 m (between river miles 311 and 312) in high discharge conditions (e.g., $Q_4$), the localized fast flow is not observed.

### Turbulence quantities

**Figure 6:**
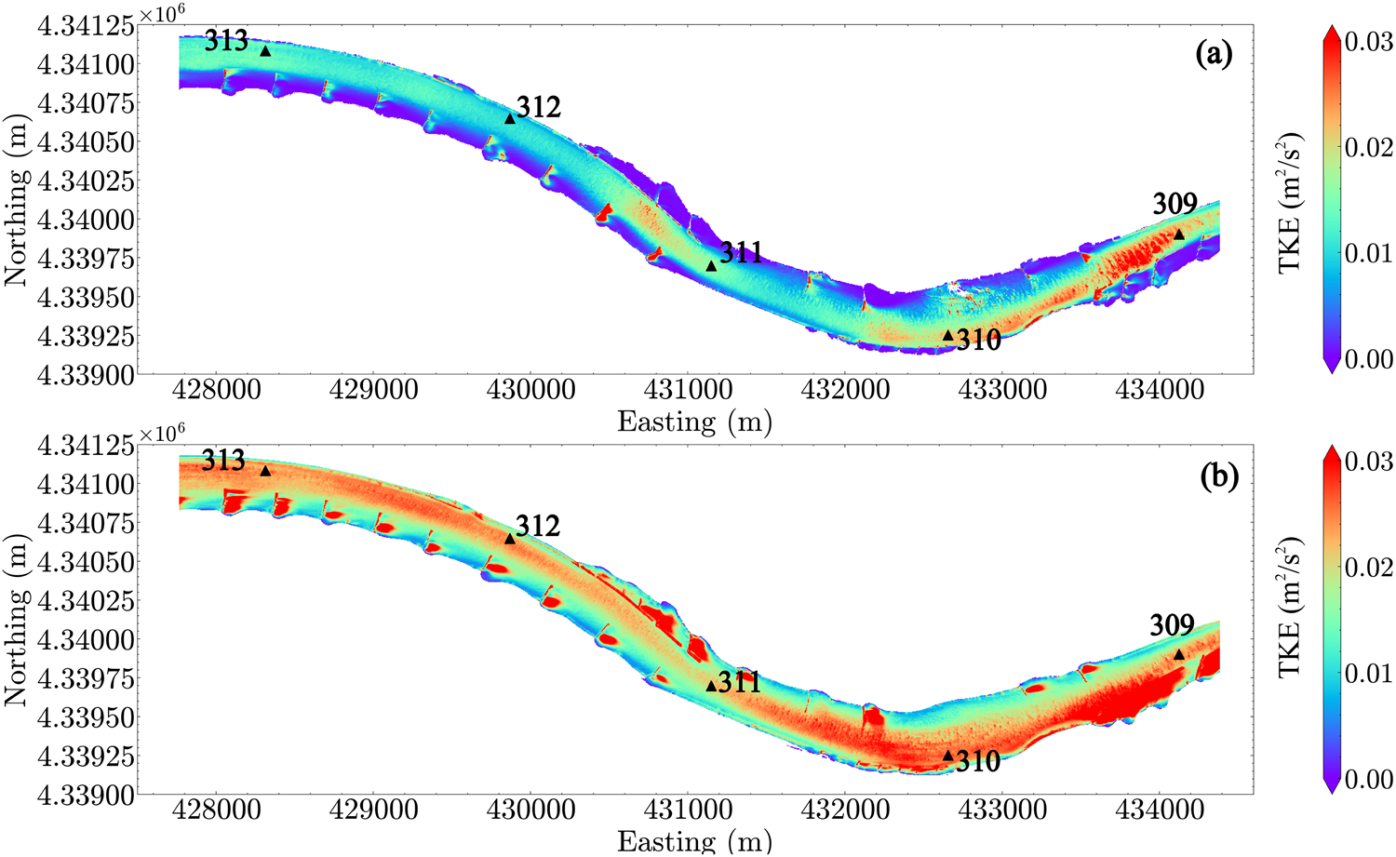
Depth-averaged turbulence kinetic energy (TKE): (**a**) $Q_1$; (**b**) $Q_4$. River miles 309–313 are indicated in the plot by black triangles.

**Figure 7:**
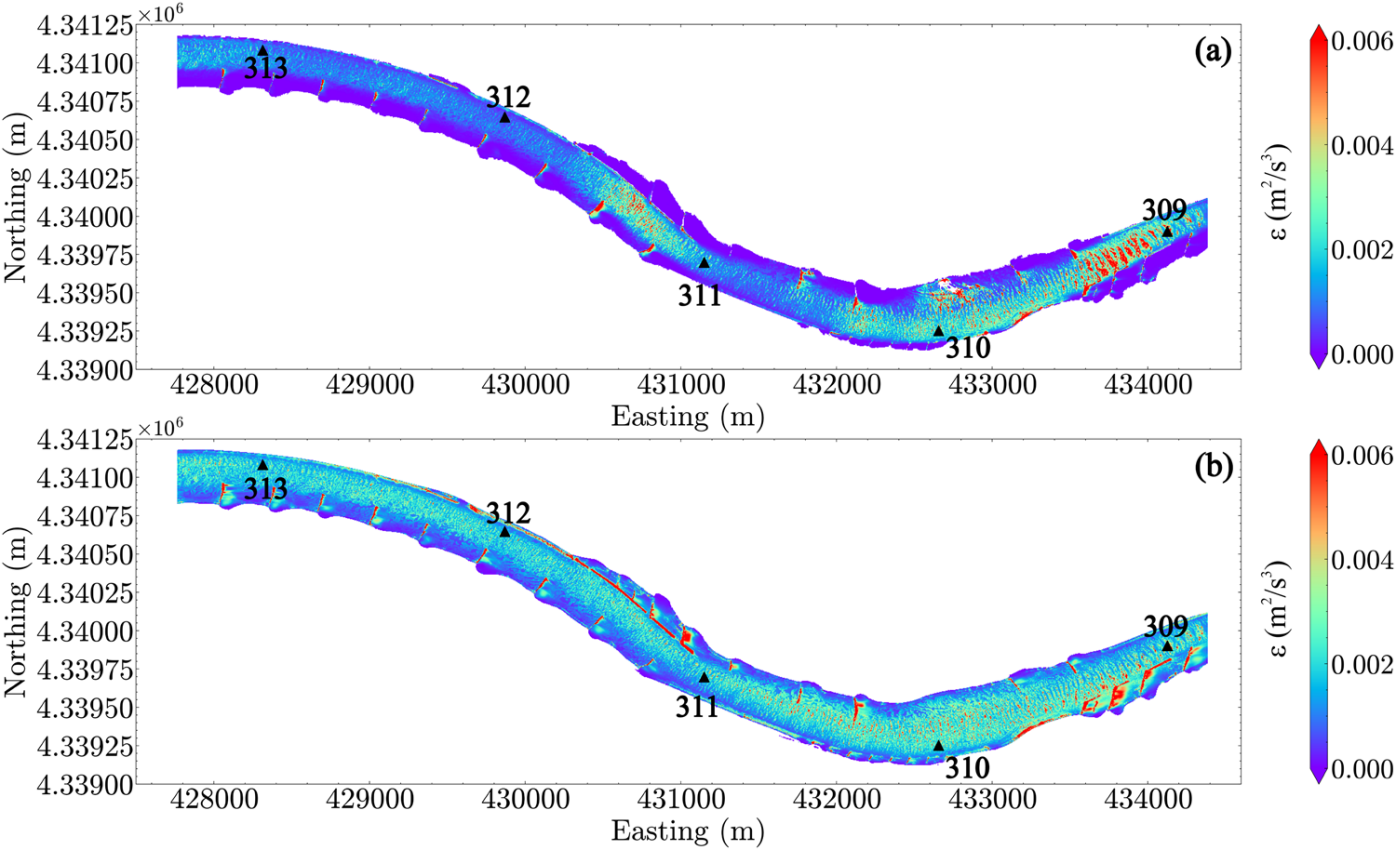
Depth-averaged turbulence dissipation rate: (**a**) $Q_1$; (**b**) $Q_4$. River miles 309–313 are indicated in the plot by black triangles.

Two turbulence quantities were selected to elucidate the turbulence in the reach: the depth-averaged TKE (Fig. 6) and the depth-averaged dissipation rate of TKE (Fig. 7). For $Q_1$, TKE shows a similar spatial pattern as the flow velocity, indicating that the high TKE is usually associated with high velocities. For $Q_4$, additional high TKE regions are located within the low velocity zones near the dikes. These high turbulence regions are caused by the interaction of flow with the hydraulic structures. For instance, enhanced turbulence may occur within wakes downstream from the flows over the dikes. Strong shear-induced turbulence may also occur at the water surface near the edge of the dikes close to the main channel. Similar to TKE, the locations of high TKE dissipation rate are coincident with high velocity in the main channel and near the dikes where strong flow-structure interactions occur.

**Figure 8:**
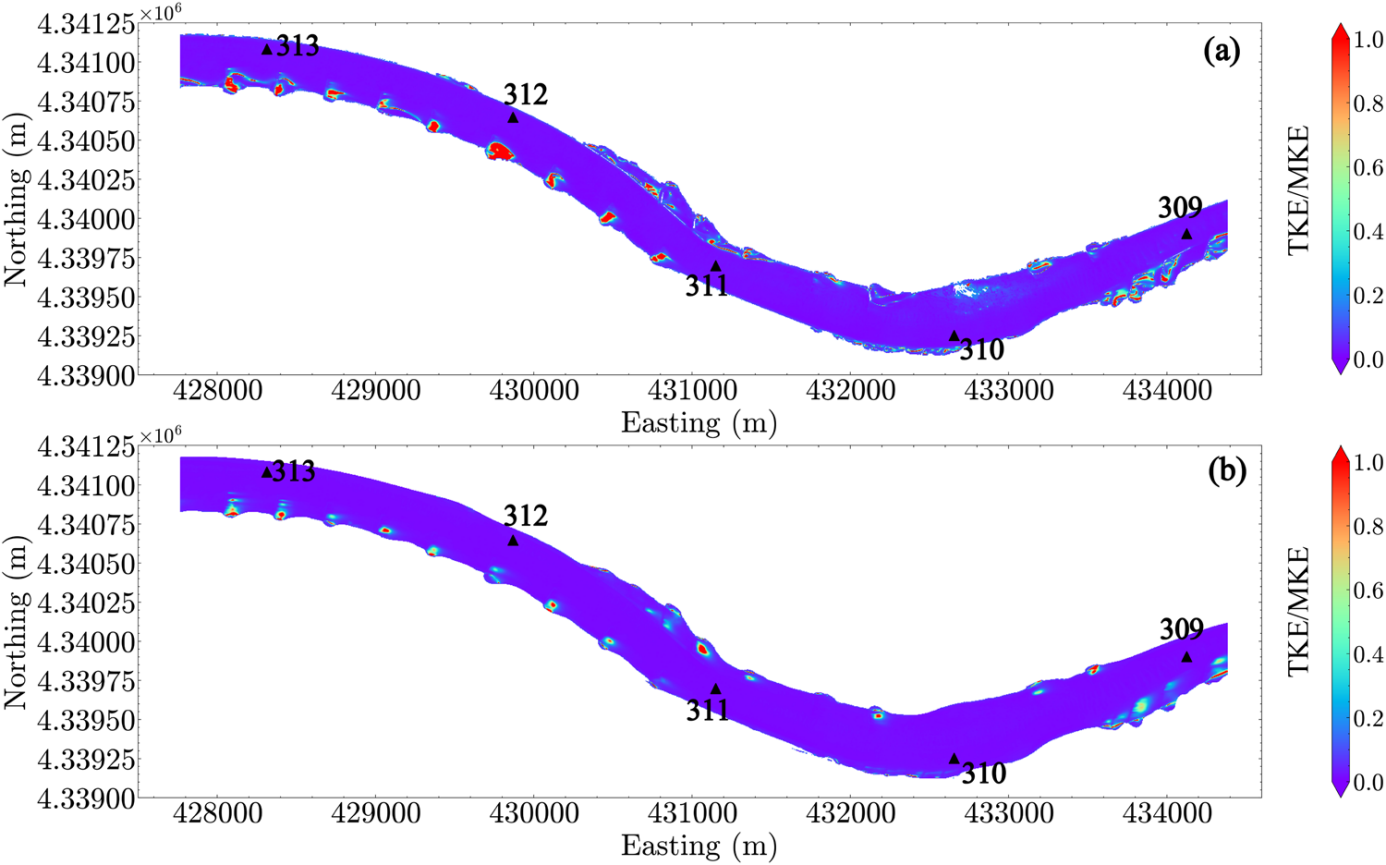
The ratio between turbulent kinetic energy (TKE) and mean kinetic energy (MKE) in the reach: (**a**) $Q_1$; (**b**) $Q_4$. River miles 309–313 are indicated in the plot by black triangles.

To examine the correlation between turbulence and the mean flow in the reach, Fig. 8 elucidates the ratio between TKE and the mean kinetic energy (MKE) where MKE is defined based on mean velocity values, MKE = $0.5(U^2+V^2+W^2)$. The data show that the TKE/MKE ratio is much smaller than 1 in the main channel, a typical open-channel feature. However, near the river bank and in the dike fields, greater TKE than MKE is common, with the spatial distribution of TKE/MKE$>1$ being dependent on discharge. This result documents strong interactions between water flow and the solid boundaries, which generate substantial turbulence comparing to the reduced mean velocity in these regions. Within these regions, particles would be expected to have longer residence times^32^.

### Egg suspension and settling

**Figure 9:**
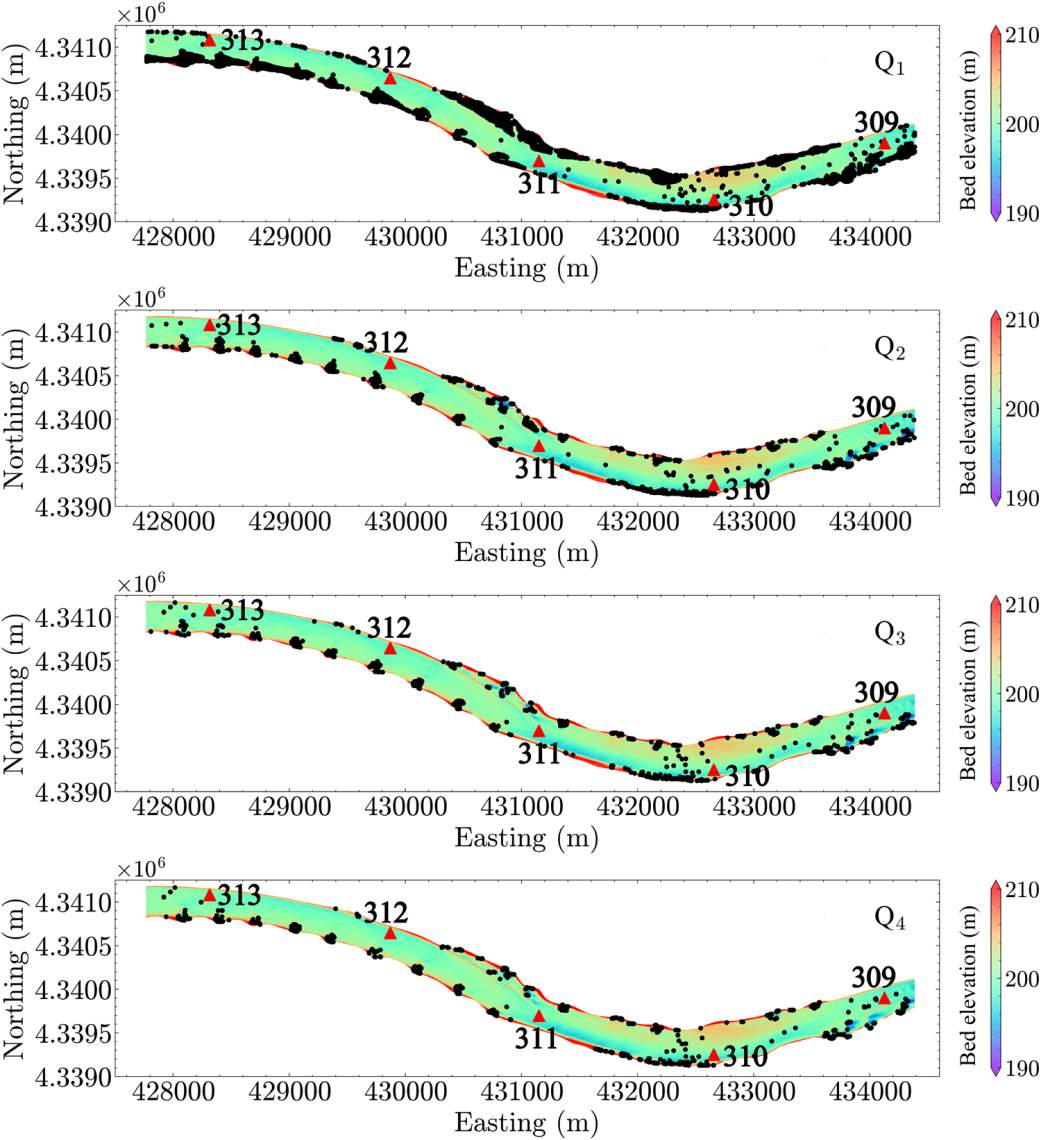
Predicted egg settling locations using the criterion of Rouse number. Black dots show the locations where the turbulence is inadequate to keep eggs suspended, i.e., inferring egg settling. Note that the egg settling is evaluated at all nodes in the three-dimensional computational fluid dynamics (CFD) simulation results. River miles 309–313 are indicated in the plot by red triangles.

The CFD modeling results allow for analysis of potential egg settling zones based on the criteria of particle suspension outlined in section “Criterion of egg suspension and settling from the hydrodynamic model”. In Fig. 9, the potential egg settling locations are plotted based on the Rouse number criterion for all four discharge conditions. The plot shows that potential settling zones are located near the river banks, in dike fields, and even in the channel at locations with strong gradients in the bed morphology. We note that the criterion was applied to all data points simulated in the CFD. Therefore, the settling zones represent the *x*–*y* locations where turbulence is inadequate to suspend eggs. Not surprisingly, the estimated potential settling zones become smaller with increasing discharge. Results using shear velocity and vertical turbulence intensity criteria show similar results, which are plotted in the supplementary file (Figs. S3 and S4).

**Figure 10:**
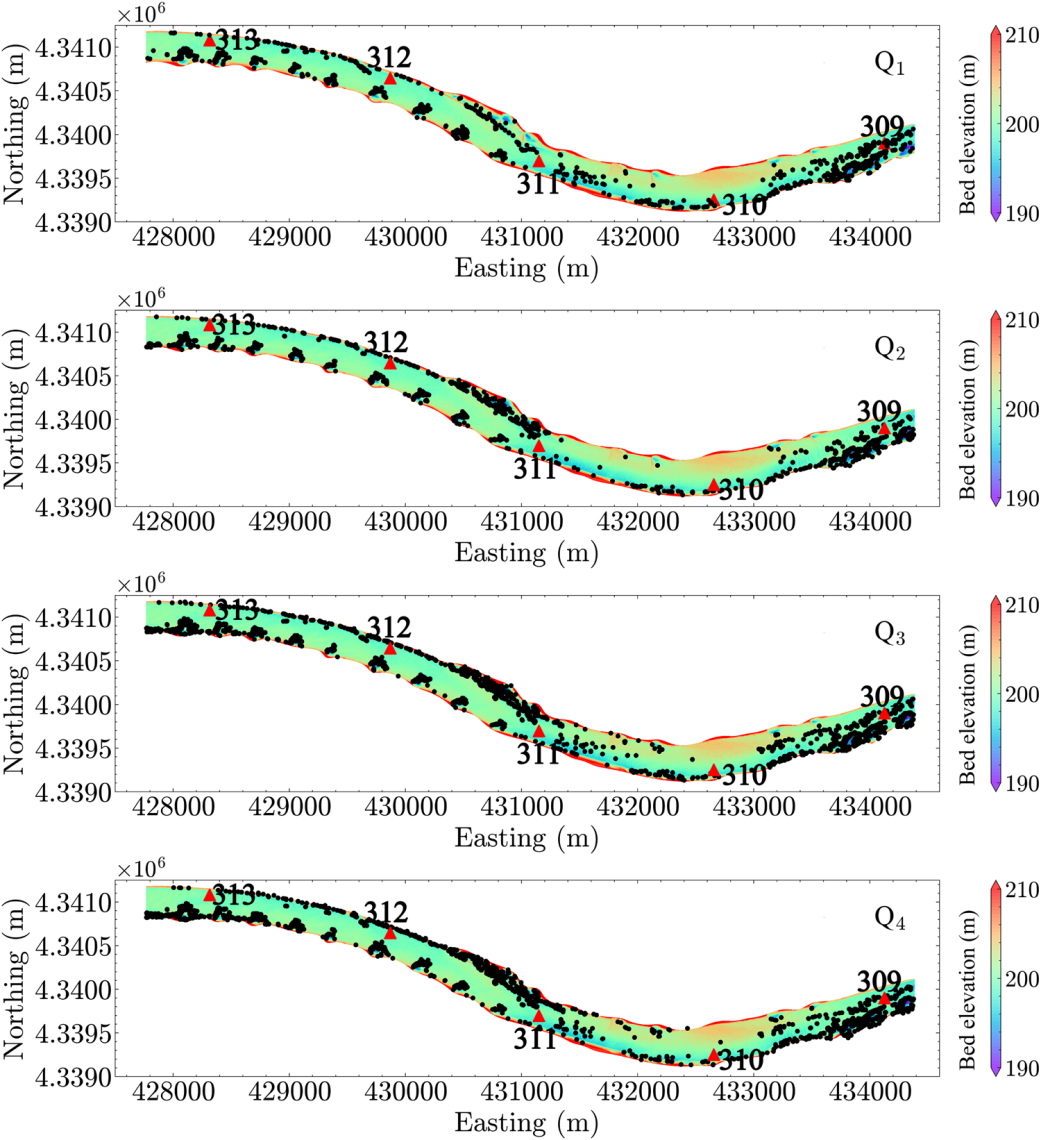
Predicted egg settling location using the egg drift model, SDrift^31^. River miles 309–313 are indicated in the plot by red triangles.

**Figure 11:**
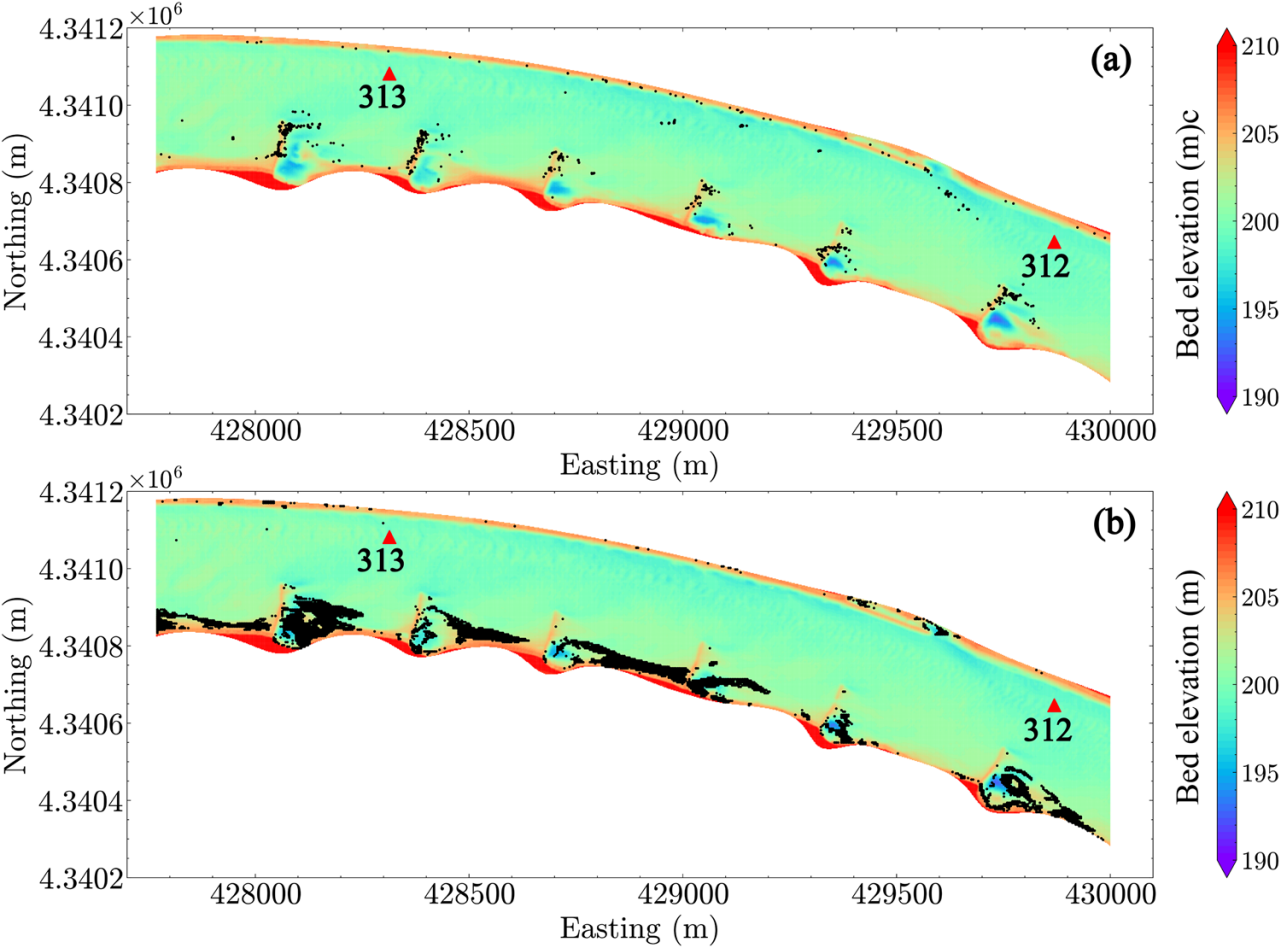
Zoom-in view of estimated egg settling zone under discharge condition $Q_1$ using (**a**) SDrift model and (**b**) hydrodynamic inference based on Rouse number criterion. River miles 312 and 313 are indicated in the plot by red triangles.

**Figure 12:**
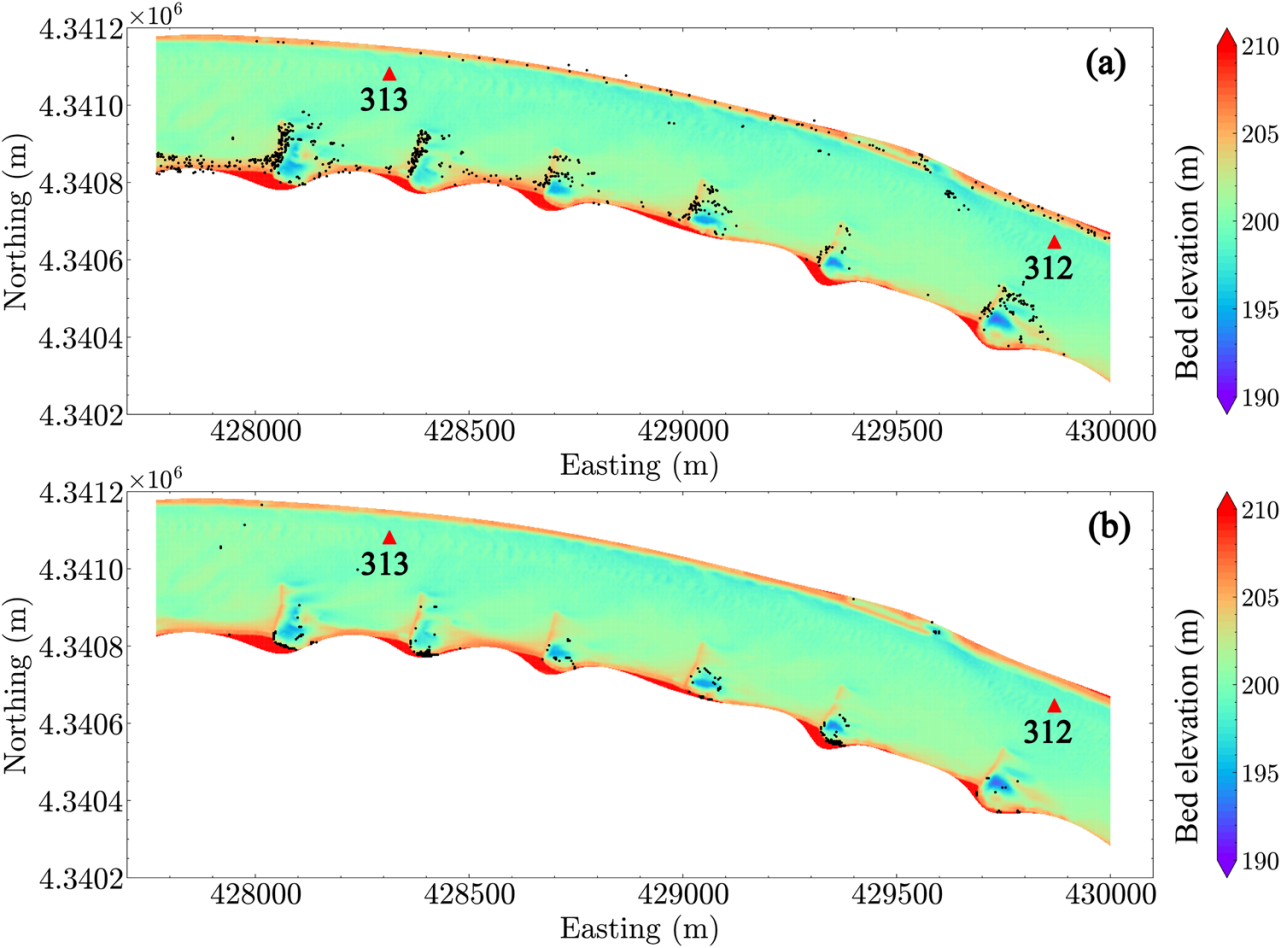
Zoom-in view of estimated egg settling zone under discharge condition $Q_4$ using (**a**) SDrift model and (**b**) hydrodynamic inference based on Rouse number criterion. River miles 312 and 313 are indicated in the plot by red triangles.

Figure 10 shows the predicted locations of entrapped eggs using the egg drift model, SDrift^31^. Comparing Fig. 10 with Fig. 9, we found that both hydrodynamic-inferred potential settling locations and drift-model predicted locations include the regions near the dike fields and the sparse areas in the channel where strong topographic gradients are present. However, careful examination of the wing dike areas (Fig. 11 under $Q_1$ condition and Fig. 12 under $Q_4$ condition), shows that the predicted egg settling zones using two methods are located in different regions near the dike areas. SDrift results indicate that egg entrapment is mainly located adjacent to the dikes, whereas the hydrodynamic inference indicates strong egg settling potential downstream from the dikes under low-flow conditions, such as the discharge condition $Q_1$ (Fig. 11). The potential egg settling zones are substantially decreased by increasing discharge (Fig. 12). SDrift results indicate that egg entrapment is primarily due to interception of egg movement due to strong topographic gradients near the dikes while being tracked in the model under these hydrodynamic conditions. Although this does not directly imply that the eggs would settle in these areas, higher probability of egg-dike interaction would occur that could potentially affect egg survival. In contrast, the hydrodynamic inference only suggests hydrodynamic conditions that are favorable for egg settling, which differs from the drift models.

In addition, the drift model predicts substantial egg entrapment near the left bank upstream of the bend located around $x =$43,100 m (upstream of river mile 311), where these regions were not inferred from hydrodynamic data. The differences indicate that eggs can be entrapped within locations where hydrodynamics would indicate suspension. The potential entrapment in the drift model is likely due to the reduction in egg-drift speed close to the left bank, which increases the probability of egg settling. In curved rivers reaches, the unevenly distributed flow in the cross section and secondary flow may push eggs towards the outer side of the channel, which can increase the probability of the particle-bank interaction.

**Figure 13:**
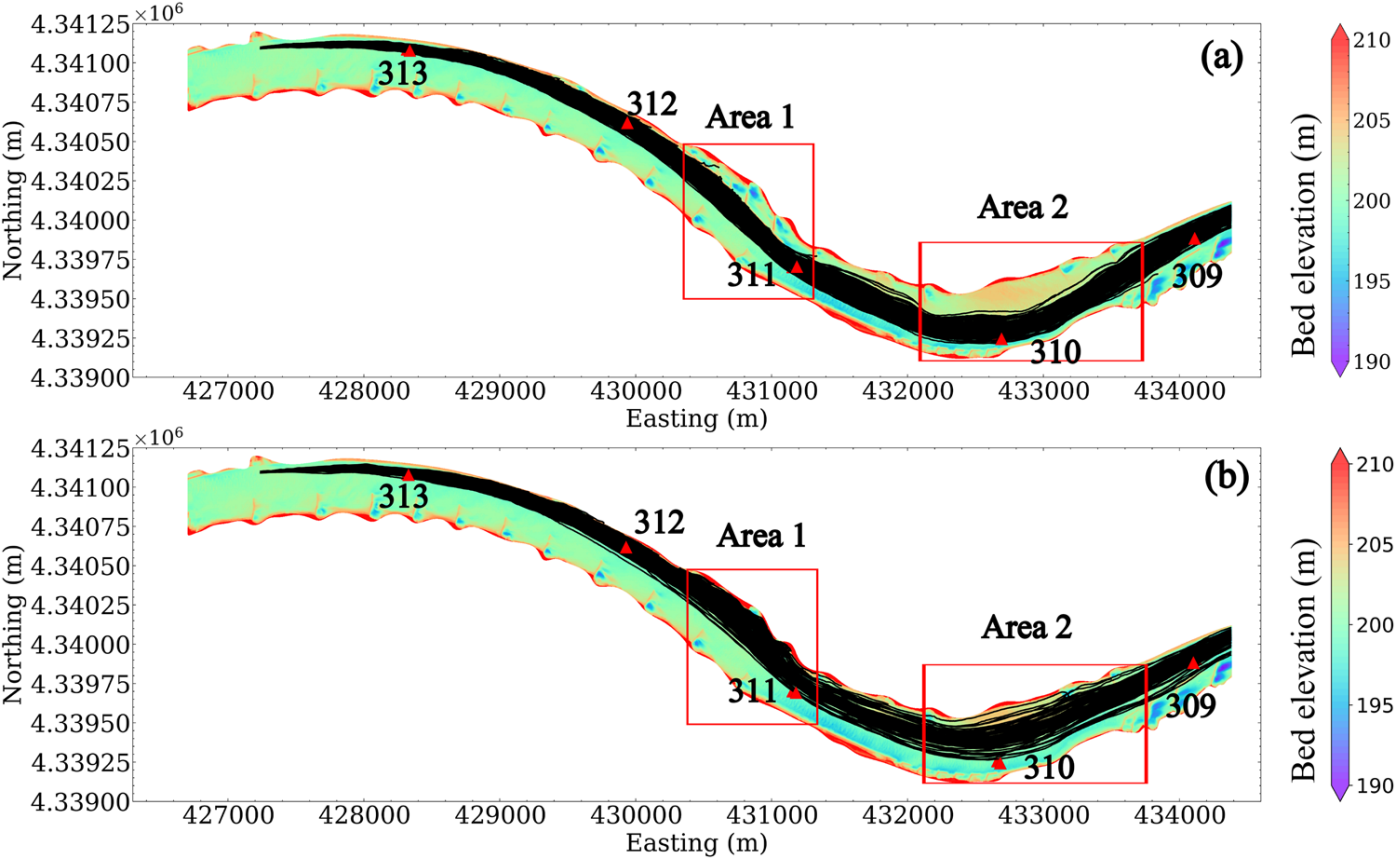
Trajectories of 200 SDrift simulated eggs near the left bank at the release point at two discharges: (**a**) $Q_1$, (**b**) $Q_4$. River miles 309–313 are indicated in the plot by red triangles.

The drift trajectories of 200 simulated eggs released near the left bank for discharge $Q_1$ and $Q_4$ can be used to visualize drift dynamics simulated in SDrift (Fig. 13). The modeling results show that, under $Q_1$, there is minimal egg drift into the low-flow region between the L-head dikes and the left bank in Area 1, as well as into the high-riverbed region close to the left bank in Area 2. This restriction occurs because the elevation of the dikes in Area 1 are higher than the water surface elevation during low-flow conditions, preventing eggs from entering these areas. As a result, the drift model predicts minimal entrapment of eggs in these areas. However, the hydrodynamic inference only takes into account favorable conditions for egg settling, implying significant settling in these regions even when trajectories would fail to transport eggs into the areas. Nevertheless, under higher-flow conditions that permit eggs to enter these areas (see Fig. 13b), particularly in Area 1, entrapment of eggs can occur (see Fig. 10), even though the hydrodynamic inference does not indicate significant settling compared to other low-velocity areas.

### Vertical distribution of potential egg settling zones

**Figure 14:**
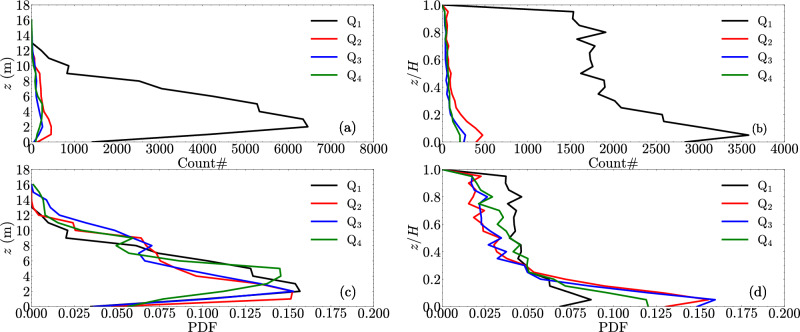
Vertical distribution of hydrodynamic-inferred egg settling locations using the criterion of Rouse number. (**a**) Number of counts as a function of different heights (*z*) above the riverbed; (**b**) number of counts as a function of the normalized heights which are normalized using flow depth (*H*); (**c**) probability distribution function (PDF) of the occurrence as a function of *z*; (**d**) PDF of the occurrence as a function of *z*/*H*.

To examine the likelihood of egg settling based on vertical position in the water column, the number of cells were counted that satisfy the criterion of egg settling based on hydrodynamic inference at the same vertical height above the riverbed (*z*) under the four simulated discharges. Figure 14 illustrates an example based on Rouse number criterion. The results show that the flow condition of $Q_1$ has substantially more counts (about one order of magnitude) due to weaker turbulence compared to the other three flow conditions (Fig. 14a and b). We interpret this large change between $Q_1$ and higher discharges as a threshold resulting when flows begin to overtop the wing dikes. Overtopping flows substantially decrease low-turbulence areas downstream and landward of wing dikes.

The modeling data also indicate that egg settling is more likely to occur in the lower part of water column but not near the riverbed. Taking $Q_1$ as an example, the peak of the number of counts are located about 2 m above the riverbed, with the number of counts decreasing both towards surface and towards the riverbed (Fig. 14a). In the normalized water column profile (Fig. 14b), substantial counts are located within the bottom 20% of the water column. We note that various water depths occur across the river reach, and hence the number of counts on the x-axis of the plots (Fig. 14a and b) are different before and after the water column normalization.

Examining the probability distribution function (PDF), we found that four discharge conditions show similar vertical profiles: egg settling has more than 10% probability within approximately the bottom 5 m (Fig. 14c), corresponding to approximately the bottom 20% of water depth (Fig. 14d). This result suggests that when eggs are transported to the bottom 20% layer, the hydrodynamic condition is less favorable for them to be re-suspended compared to higher-up in the water column. Similar results of profiles were found for the criterion using shear velocity and the vertical turbulence intensity, albeit the number of counts and the PDF values are different due to different criteria (see supplementary file, Figs. S5 and S6).

### Discussion on the egg survival

Examining river hydrodynamics in three dimensions through well-calibrated models yields valuable insights into the spatial distribution of flow velocity, water depth, and associated turbulence. These parameters can be used to identify potential locations where carp eggs may settle. However, using and interpreting results based on hydrodynamic criteria must be exercised with careful consideration. For instance, the Rouse number classification for particle suspension involves a broad range of values. In this study, we adopted $Ro > 1.2$ as an indicator of egg settling, with $Ro = 1.2$ representing the lower Rouse number bound for partial suspension. Conservatively, a critical value of $Ro = 2.5$^23^ is recommended for assessing predominantly bedload particle transport, indicating minimal to no suspension in the water column. Hence, at Rouse numbers between 1.2 and 2.5, partial suspension would be expected. In addition, the analysis using three-dimensional drift model results indicates that carp eggs would not drift into the egg settling zones within the L-head dikes and left bank (Area 1 in Fig. 13), for example, which would have predicted settling using hydrodynamic inference under the low-flow condition. This is because the actual egg drift pathway is governed by various parameters including egg spawning locations, streamlines of water flows, and interactions of flow and hydraulic structures. Consequently, predictions relevant to invasive carp management would improve when using the hydrodynamic-inferred egg settling zones if these additional parameters were taken into account.

Although egg settling zones based on hydrodynamic inference may not represent the actual conditions for egg settling, those predictions provide valuable information about the local hydrodynamics and suitability for egg settling at lower computational cost compared to drift modeling (for example SDrift). Therefore, this information could be useful for managers in determining the desirability of implementing hydraulic controls for egg settling. For example, if flow patterns can be adjusted to guide eggs into low-turbulence zones with adequate residence time, the hydrodynamics would facilitate the desired settling of eggs, aligning with management objectives for controlling aquatic invasive species. However we noted that solely using hydrodynamic inference may be misleading in invasive carp management without knowledge of drift pathways.

While high turbulence zones are the necessary environment for carp eggs to be suspended, eggs can be damaged or killed if turbulence exceeds a certain threshold. Prada et al.^43^ found an increased mortality in drifting grass carp eggs when exposed to turbulence with TKE greater than 2 m^2^/s^2^ for 1 minute in a grid-stirred turbulence tank. When TKE reaches 2.7 m^2^/s^2^, the mortality rate increased by nearly 30%. The corresponding maximal shear stresses were found to be 20 and 30 N/m^2^ near the grid for these two TKE values respectively. From our hydrodynamic model, mean TKE in the simulated reach under discharges $Q_1$ to $Q_4$ ranges from 0.01 to 0.02 m^2^/s^2^, with maximal depth-averaged TKE ranging from 0.16 to 0.21 m^2^/s^2^. The maximal TKE in the water column is found within 0.31–0.38 m^2^/s^2^ under four discharge conditions. These values are much smaller than the reported values that are harmful for carp eggs. Therefore, in a typical egg drift process, it is unlikely for eggs to experience persistent, extreme turbulence that could cause direct damage or mortality.

However, strong turbulence often generates high suspension and transport of sediment in the river. The abrasion between carp eggs and the suspended sediment may affect the egg survival rate. In the laboratory experiment conducted by Prada et al.^15^, carp eggs were found to drift within the lower 75% of the water column with lower flow velocity in the flume (0.08 m/s). When the flow velocity was increased to 0.22 m/s, the egg distribution in the water column was uniform, indicating a well-suspended condition for carp eggs. With further increasing flow velocity, Prada et al.^15^ observed that eggs were drifting more towards the bottom where they collided with the sediment particles. This indicates that the suspension of sediment could affect the vertical distribution of suspended eggs. They also observed reduced survival rate in medium and high flows compared to the control, while the survival rate was almost the same in low flow compared to the control. They also observed different larvae behaviors in different flow velocities, which may also contribute to the survival of carps. In our simulated Missouri River reach, the river turbulence may not pose a threat to carp eggs, but the suspended sediment could have negative effects. There has been limited study on the quantitative effects of sediment abrasion on egg mortality, indicating a fruitful subject for future studies.

## Conclusions

In this study, we analyzed the simulated hydrodynamics of an 8-km reach in the Lower Missouri River, a site characterized by extensive channelization and river training. Four discharges representing 45–3% daily flow exceedance were examined. Calibration and validation of the simulations were conducted based on field observations. Flow depth, mean flow velocity, and turbulence quantities were investigated through computational fluid dynamics modeling. Simulated results show highly varied spatial distributions of mean flow and turbulence characteristics, primarily attributed to the curvature of the channel, variation in bed morphology, and the presence of river-training hydraulic structures, including wing dikes and L-head dikes.

To investigate the use of hydrodynamics for inferring the settling and suspension of carp eggs, we applied three criteria established in previous carp egg studies to analyze the spatial distribution of potential settling zones. The simulation results enabled the identification of low turbulence zones where insufficient suspension may hinder carp egg development. When comparing these hydrodynamic-inferred egg settling zones with the entrapment predicted by a Lagrangian egg-drift model, we observed that egg drift paths significantly influenced the locations where eggs may settle or be intercepted by in-stream hydraulic structures. Therefore, it is crucial to consider additional factors, such as spawning locations and drift paths, when using hydrodynamic inference to identify potential egg settling zones and larval nursery locations for invasive carp management.

Lastly, river turbulence may also influence carp egg survival through shear stresses and interactions with suspended sediment. Our data indicate that turbulence kinetic energy in the river does not surpass the laboratory-identified threshold associated with direct egg damage. However, abrasion from suspended sediment and the complex interactions between eggs and hydraulic structures, riverbed, and banks, accentuated by high morphological variations as demonstrated in the entrapment areas in the egg drift model, could affect the overall survival rate of carp eggs.

### Supplementary Information

Supplementary Figures.

## Data Availability

The data of field measurements and modeling are available in the online repository doi:10.5066/P9X5M3WH^33^.
