# Supplementary material for: Ecological inferences on invasive carp survival using hydrodynamics and egg drift models
Source: Sci Rep. 2024 Apr 25;14:9556. doi: 10.1038/s41598-024-60189-1 (PMC11045858; doi:10.1038/s41598-024-60189-1)
Supplement: Supplementary file 1 — Supplementary Figures. [file 41598_2024_60189_MOESM1_ESM.docx]

**Ecological inferences on invasive carp survival using hydrodynamics and egg drift models**

Ruichen Xu^1^, Duane C. Chapman^2^, Caroline M. Elliott^2^, Bruce C. Call^2^, Robert B. Jacobson^3^, Binbin Wang^1,4^

^1^Department of Civil and Environmental Engineering, University of Missouri, Columbia, MO, 65211, United States

^2^U.S. Geological Survey, Columbia Environmental Research Center, Columbia, MO, 65211, United States

^3^School of Natural Resources, University of Missouri, Columbia, MO, 65211, United States

^4^Missouri Water Center, Columbia, MO, 65211, United States

Any use of trade, firm, or product names is for descriptive purposes only and does not imply endorsement by the U.S. Government.

Q = 2282 m^3^/s, cross section #1


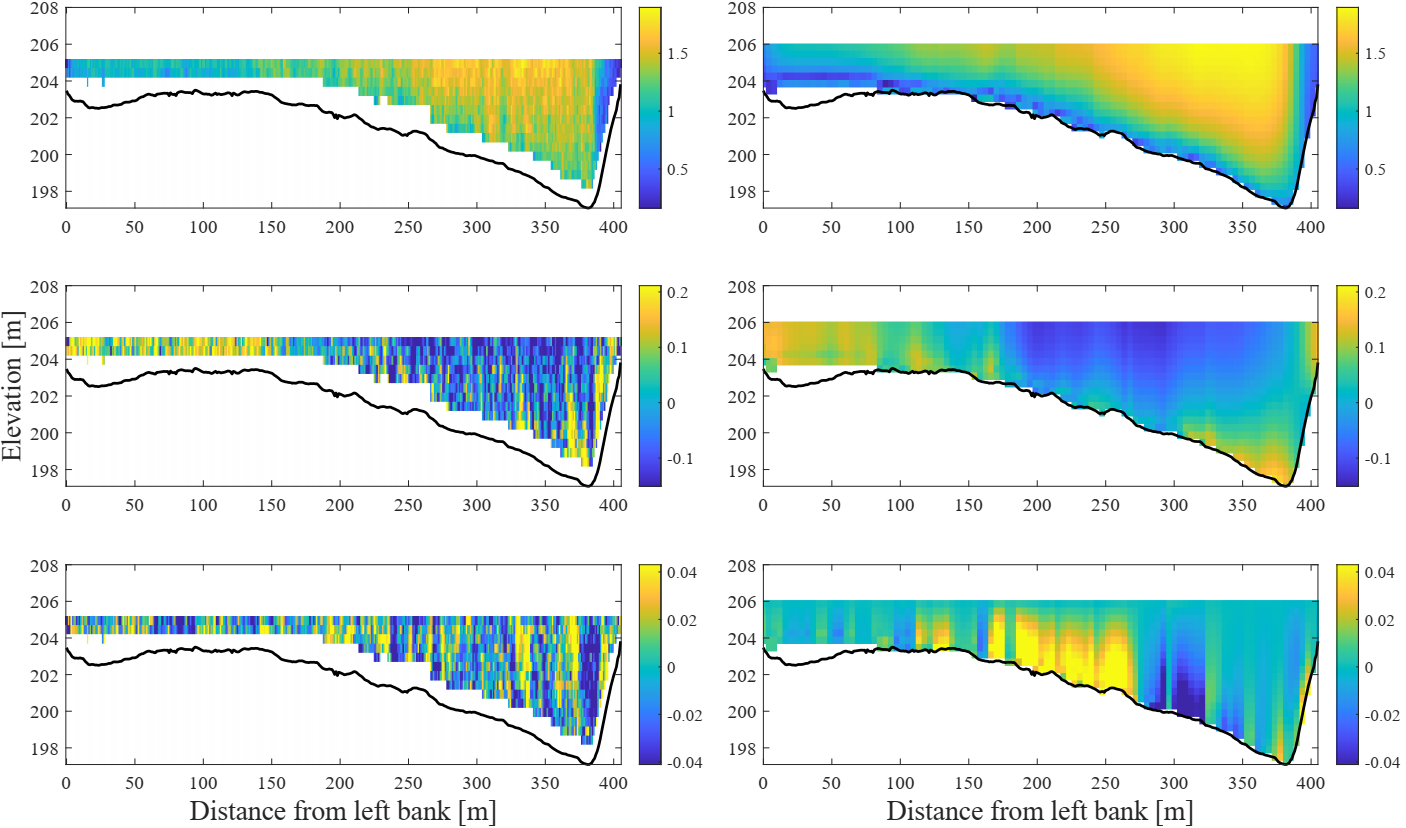


Q = 2282 m^3^/s, cross section #2


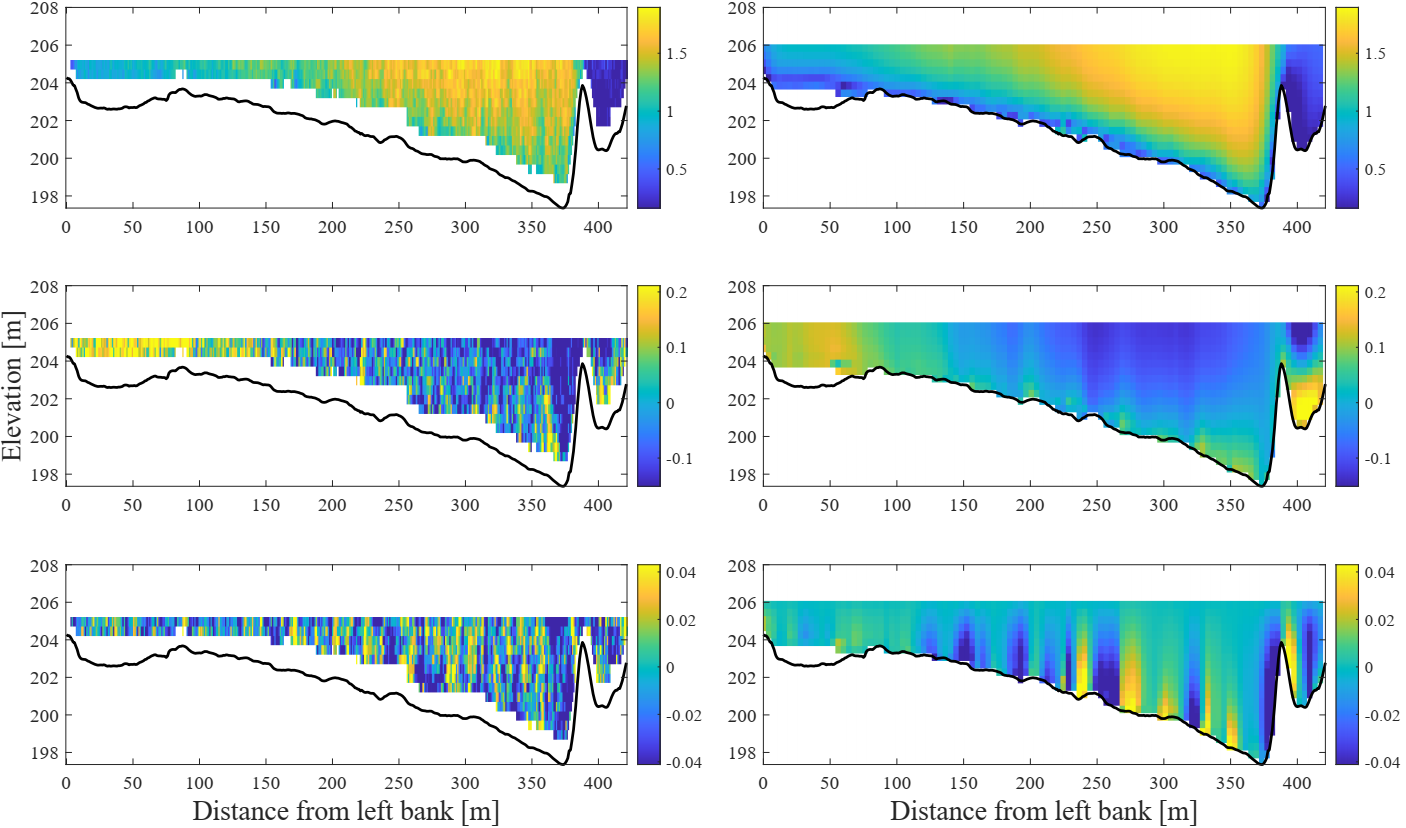


Figure S1. Comparison between model and measurement for velocity (left column: measurement; right column: modeling; first row: streamwise velocity, second row: transverse velocity, third row: vertical velocity).

Q = 2282 m^3^/s, cross section #3


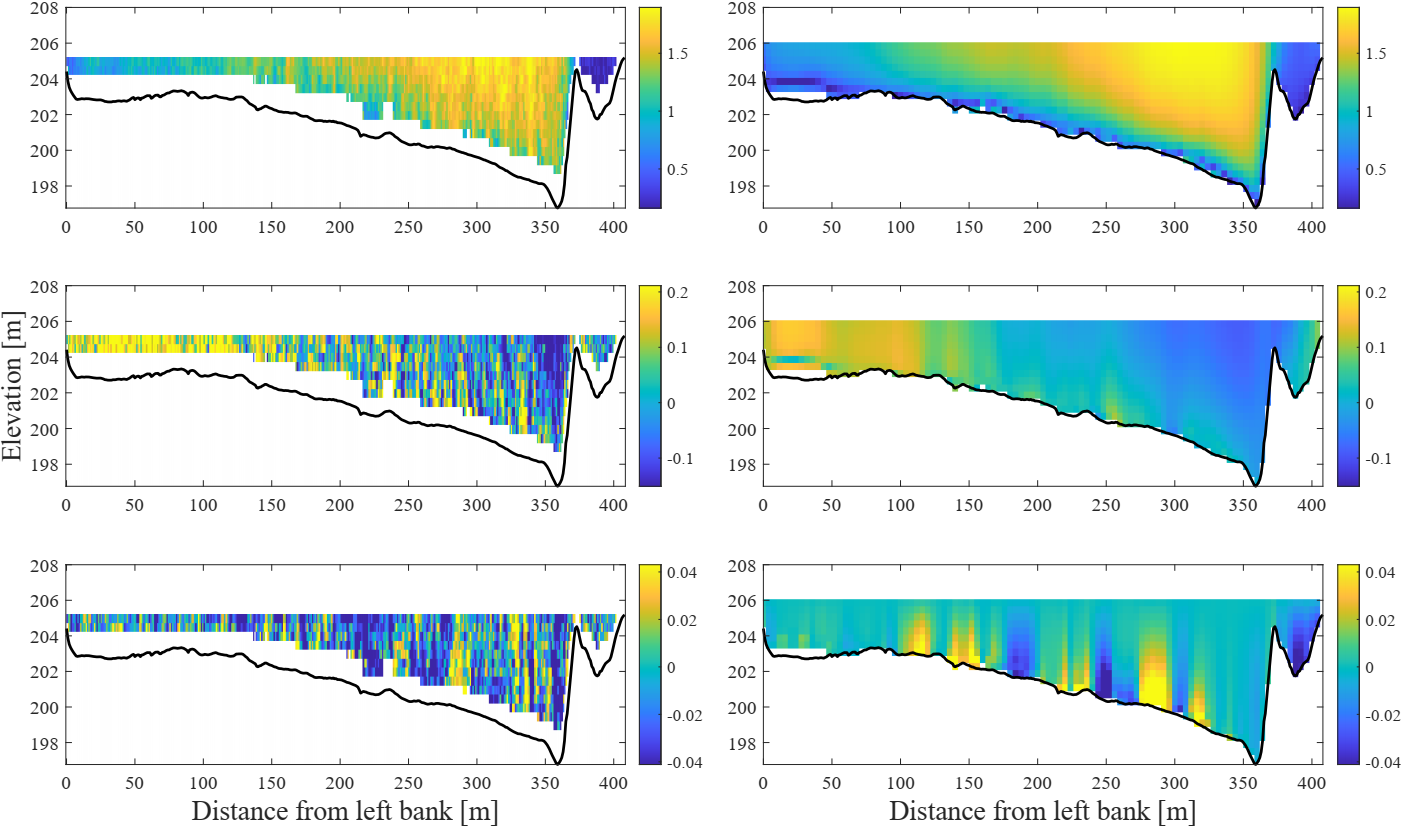


Q = 2282 m^3^/s, cross section #4


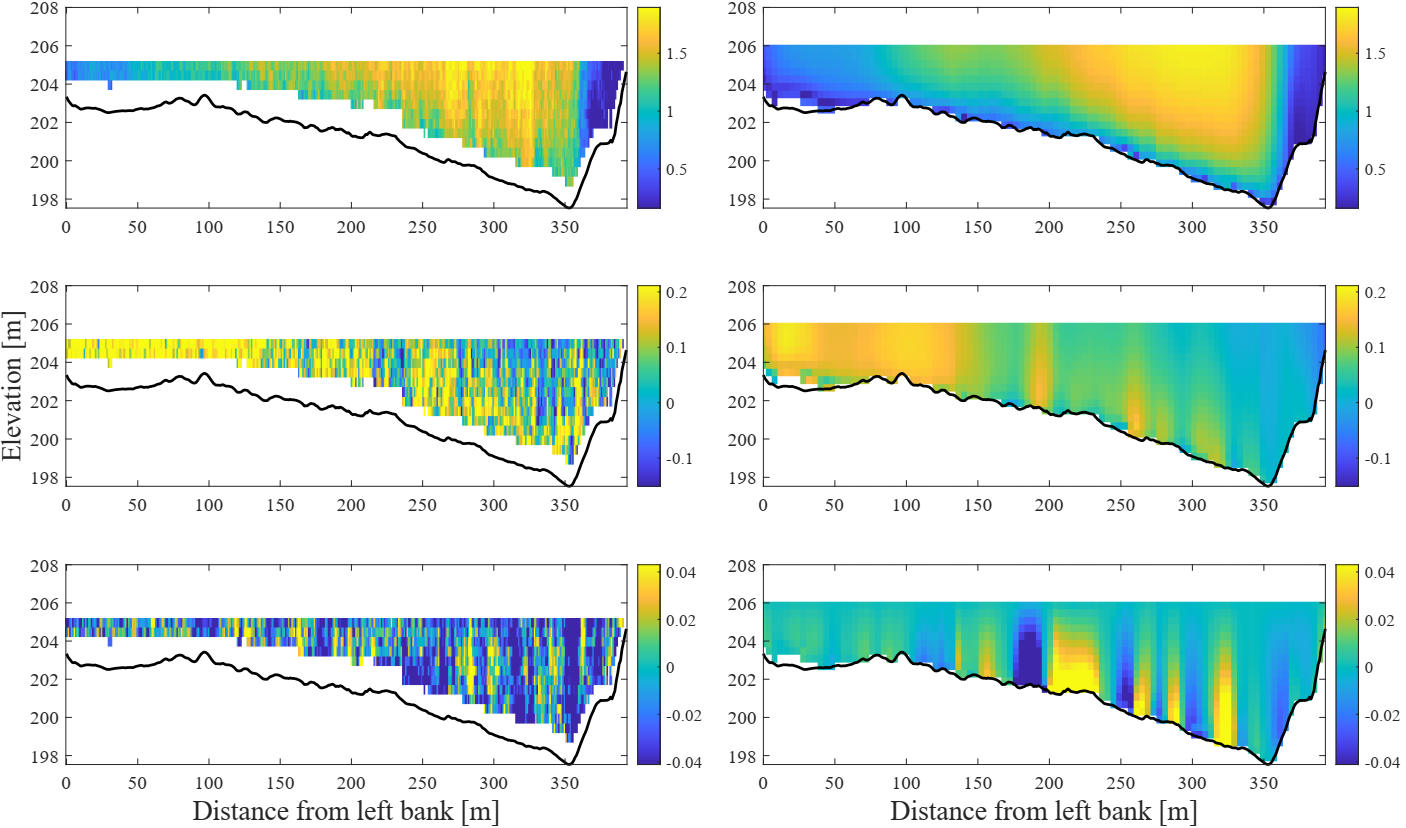


Figure S1. Continued.

Q = 2282 m^3^/s, cross section #5


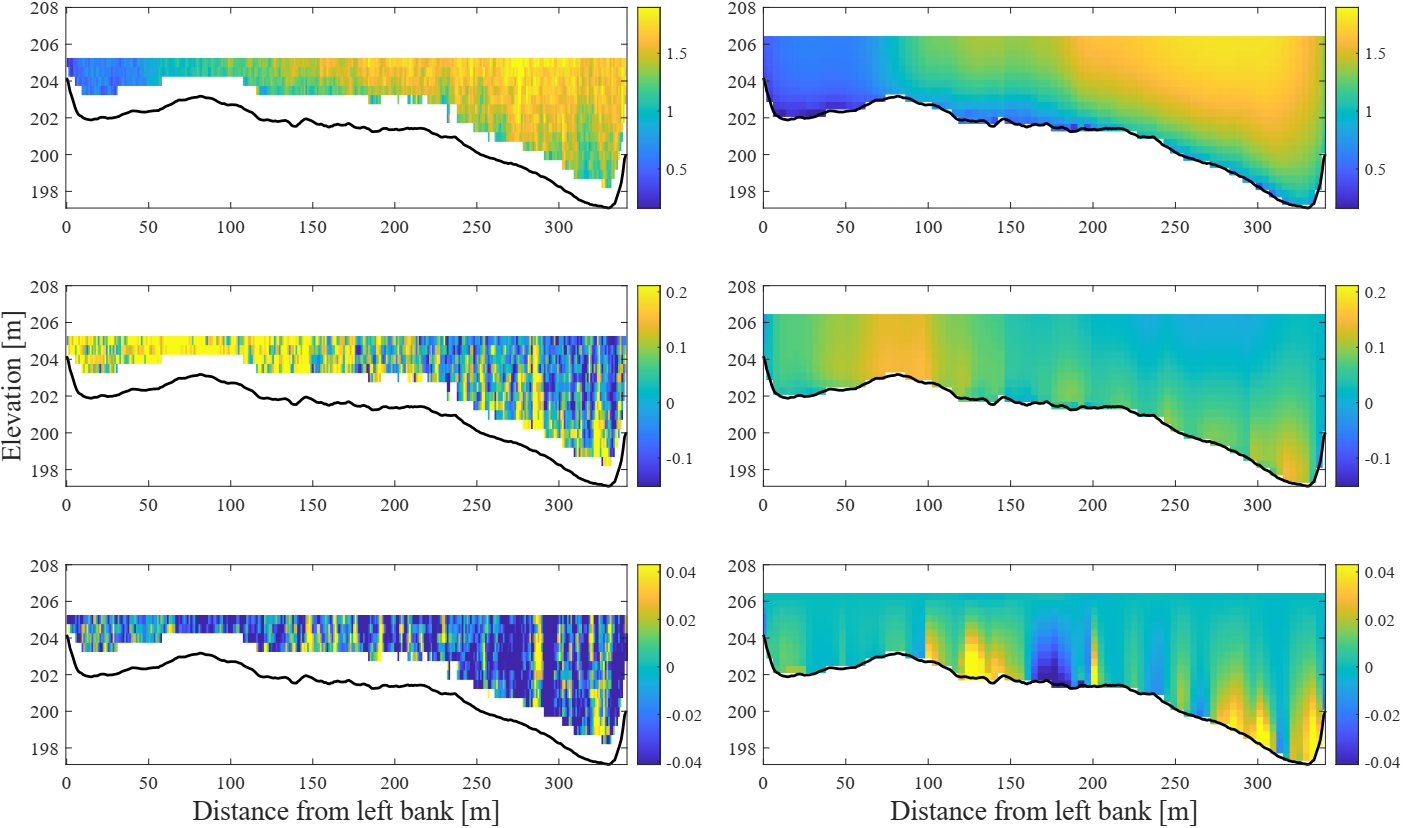


Q = 2282 m^3^/s, cross section #6


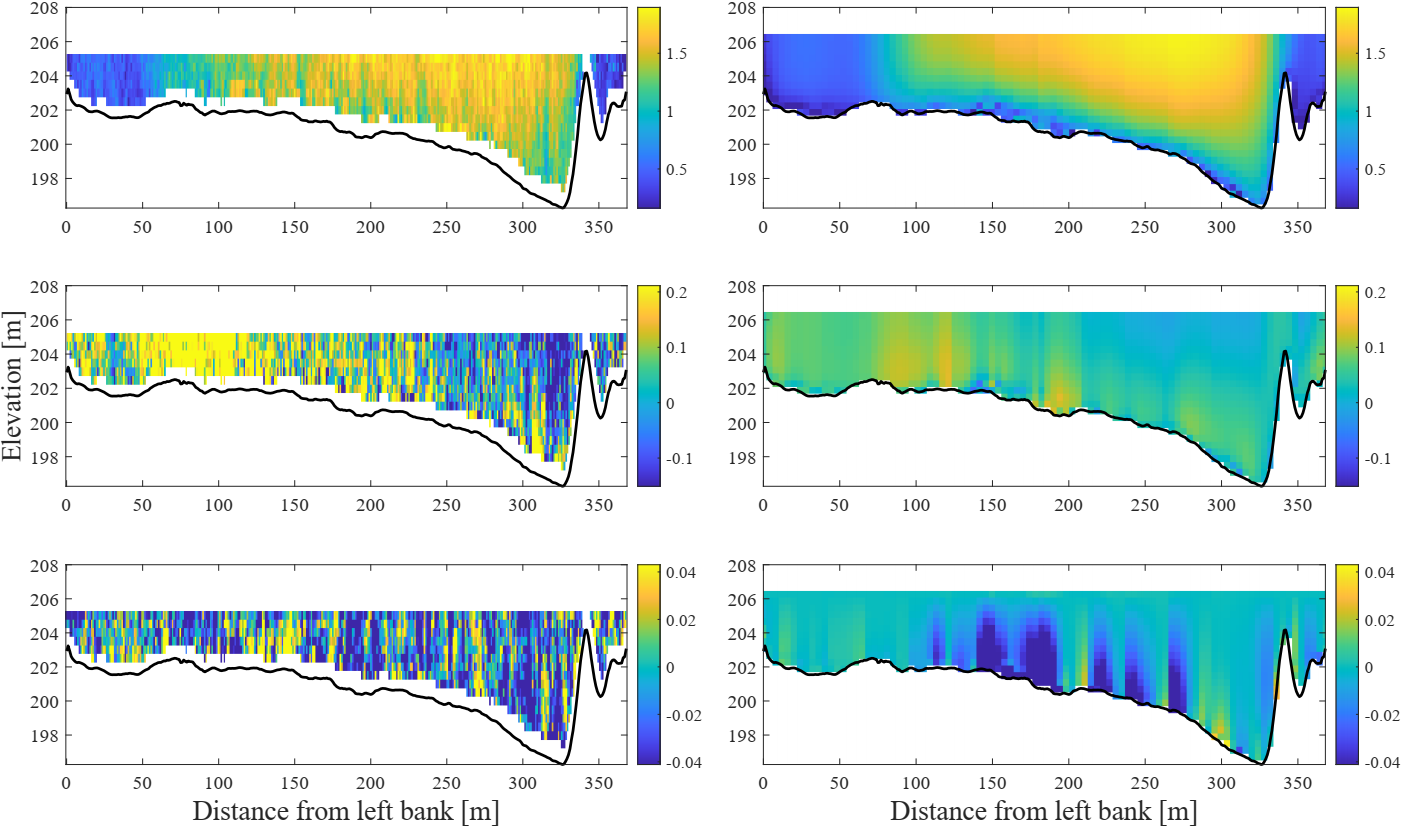


Figure S1. Continued.

Q = 2282 m^3^/s, cross section #7


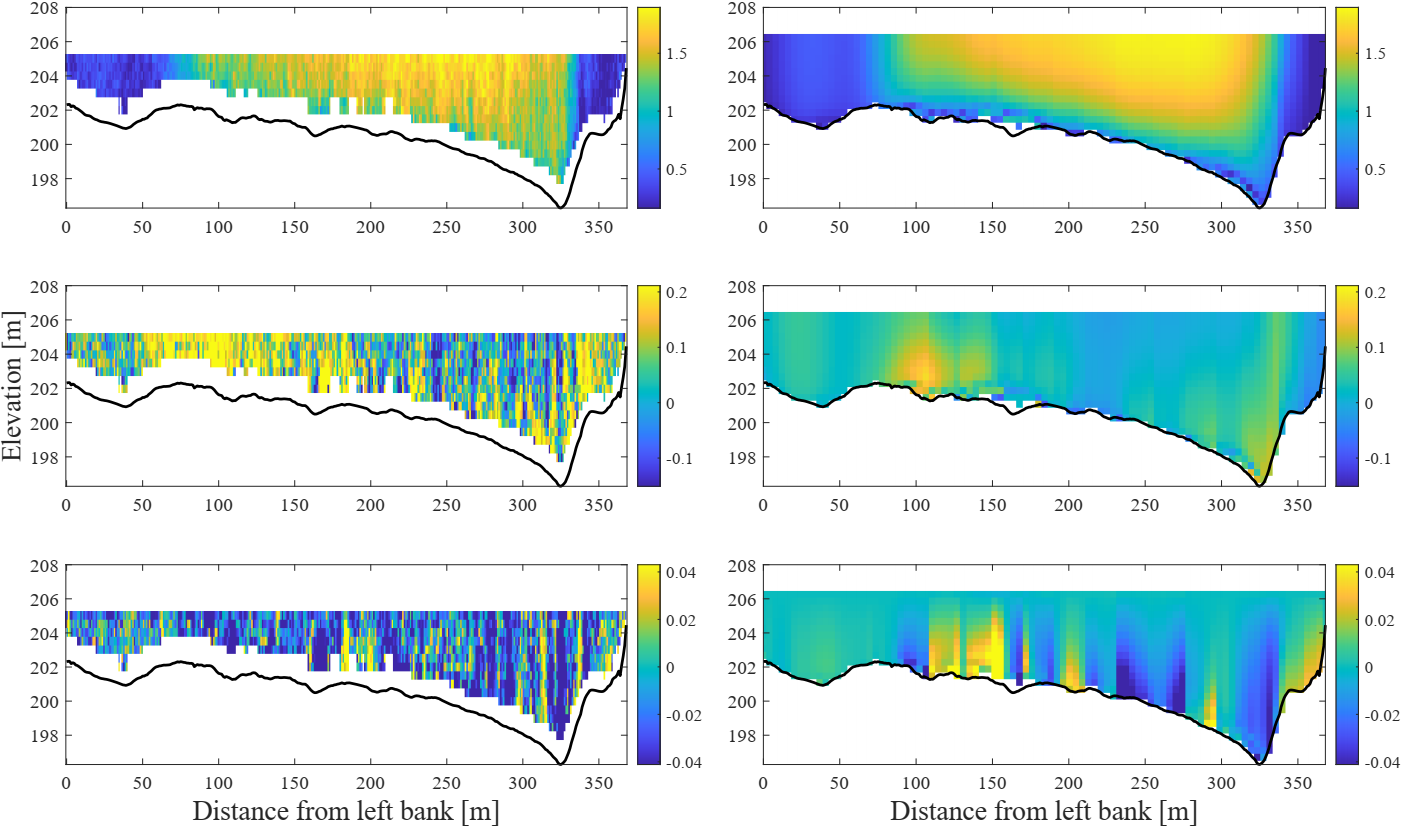


Q = 2282 m^3^/s, cross section #8


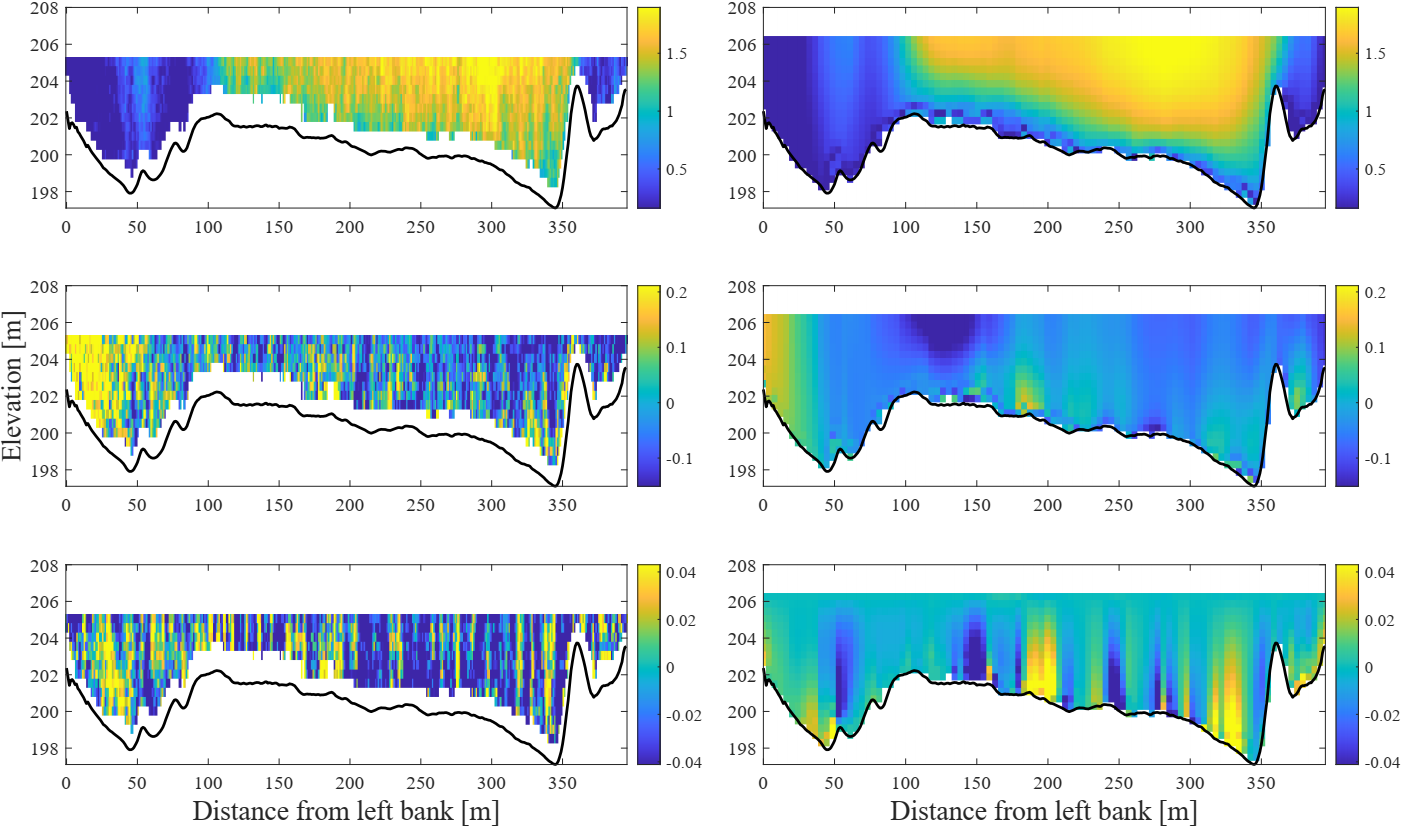


Figure S1. Continued.

Q = 2282 m^3^/s, cross section #9
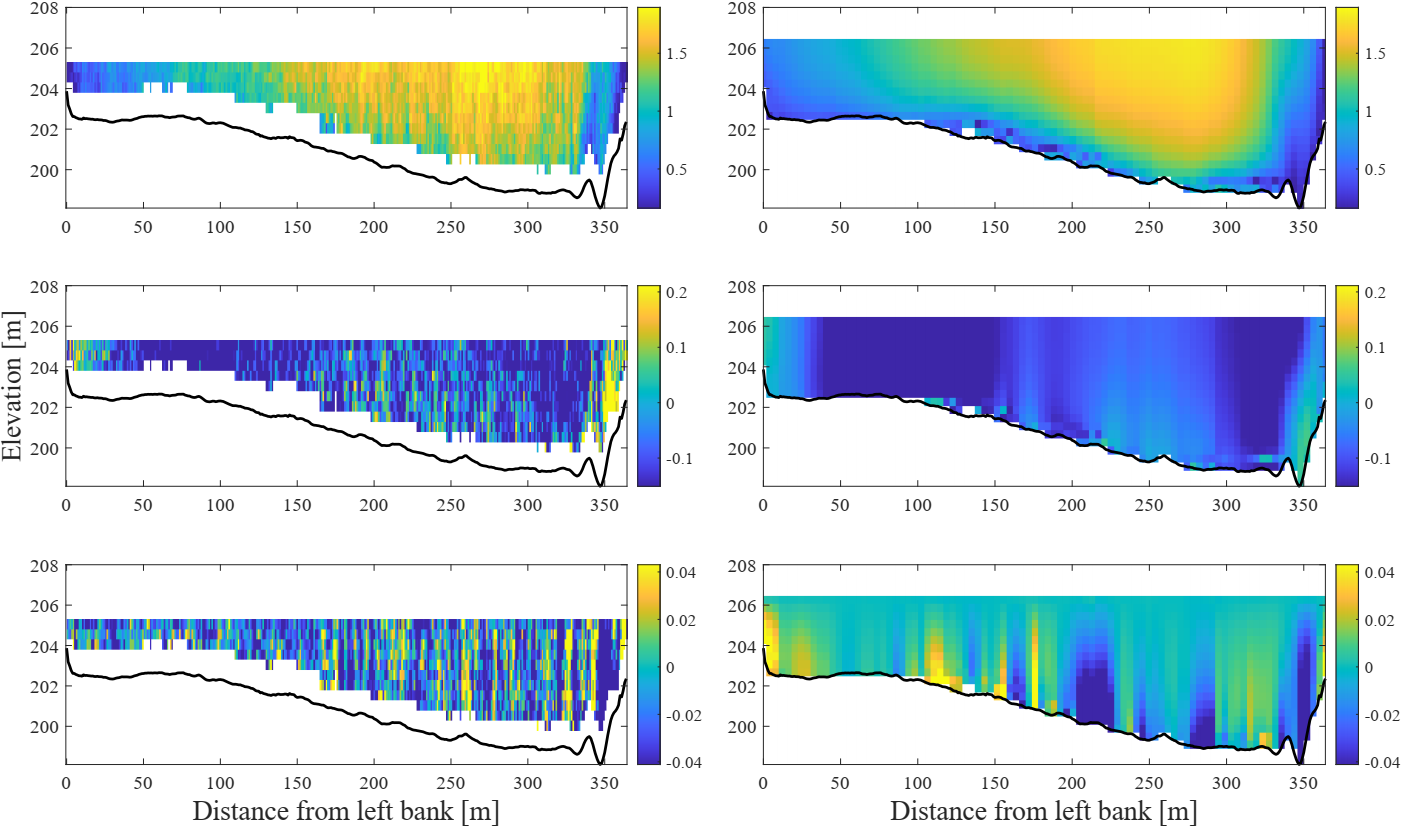


Q = 2282 m^3^/s, cross section #10
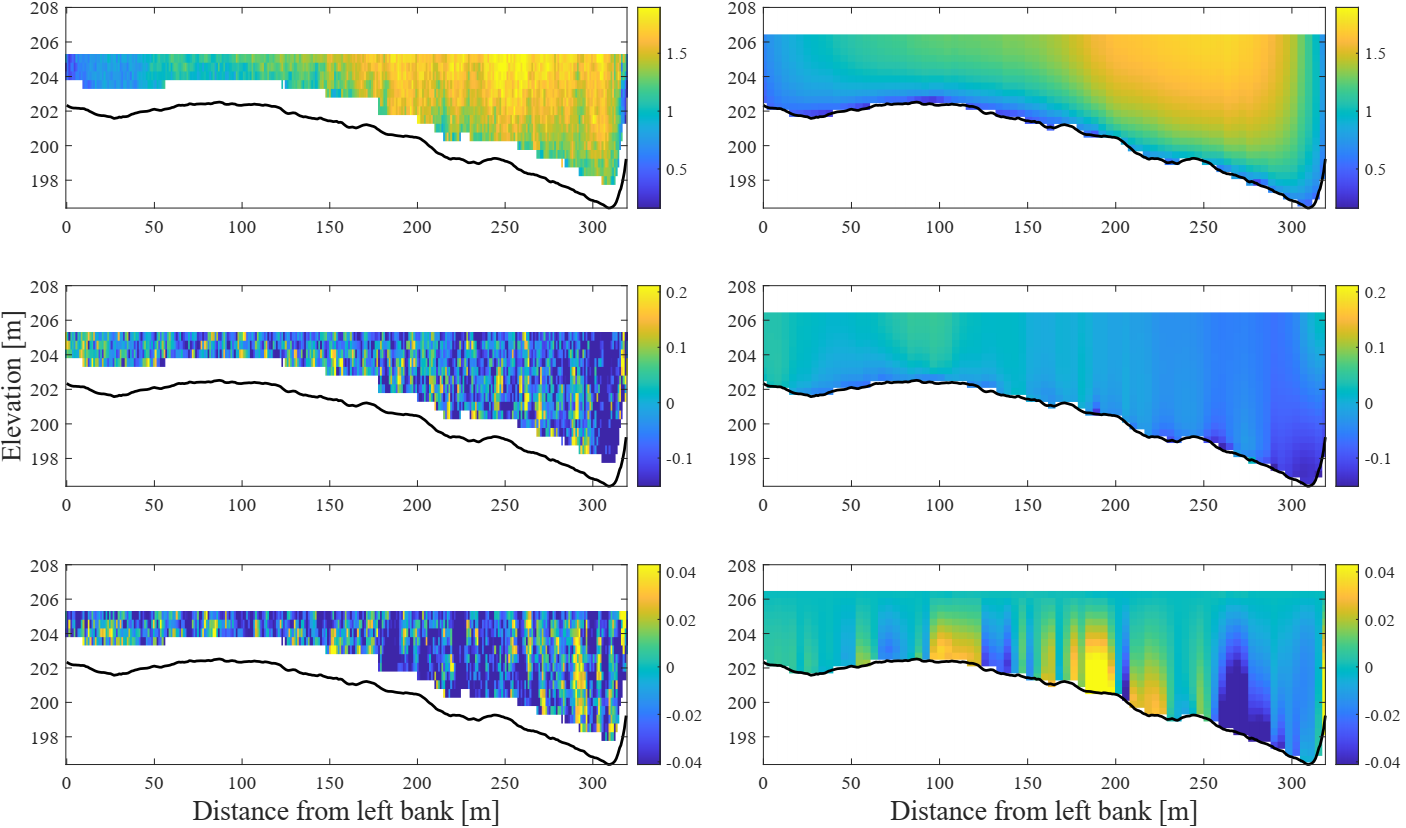


Figure S1. Continued.

Q = 2282 m^3^/s, cross section #11
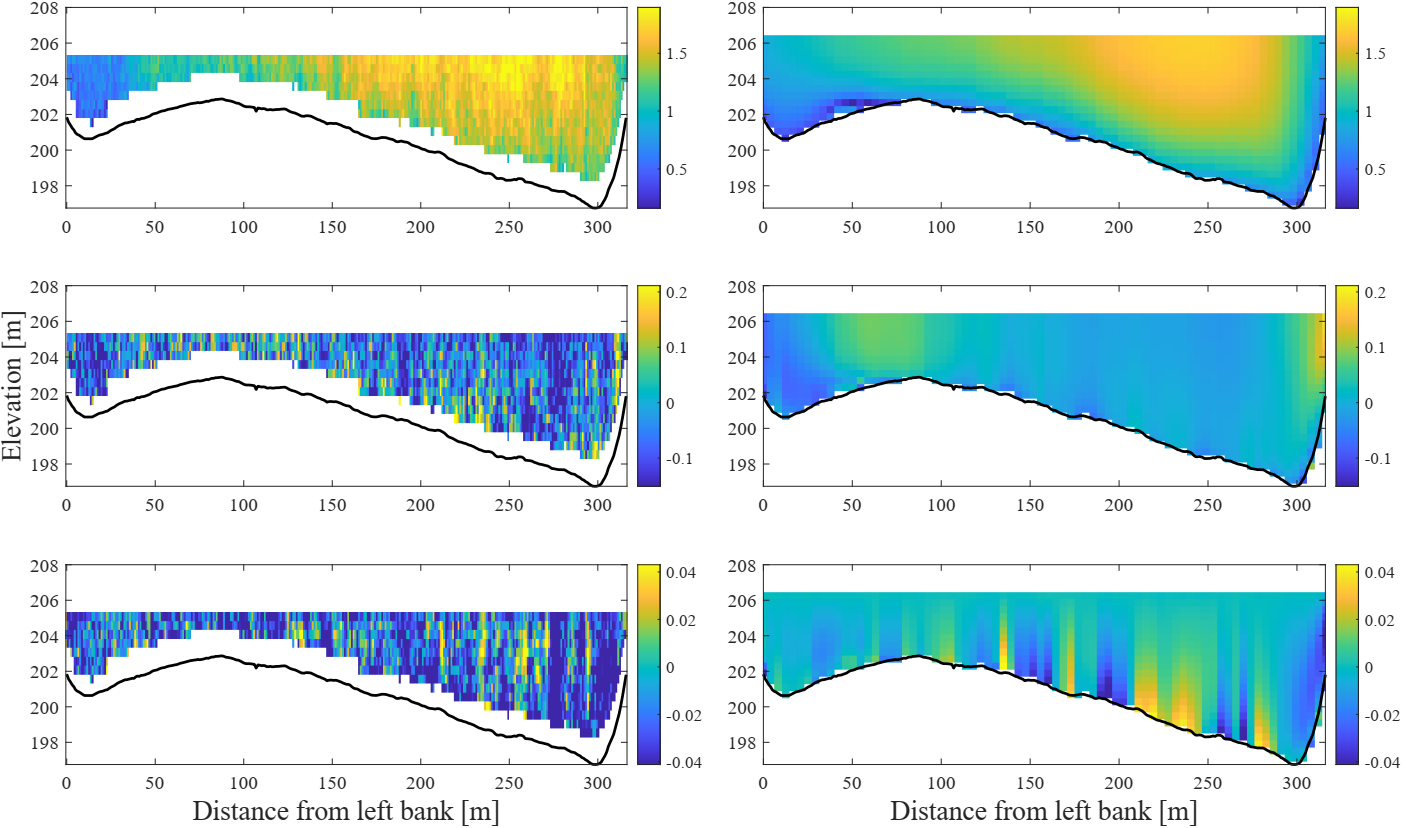


Q = 2282 m^3^/s, cross section #12
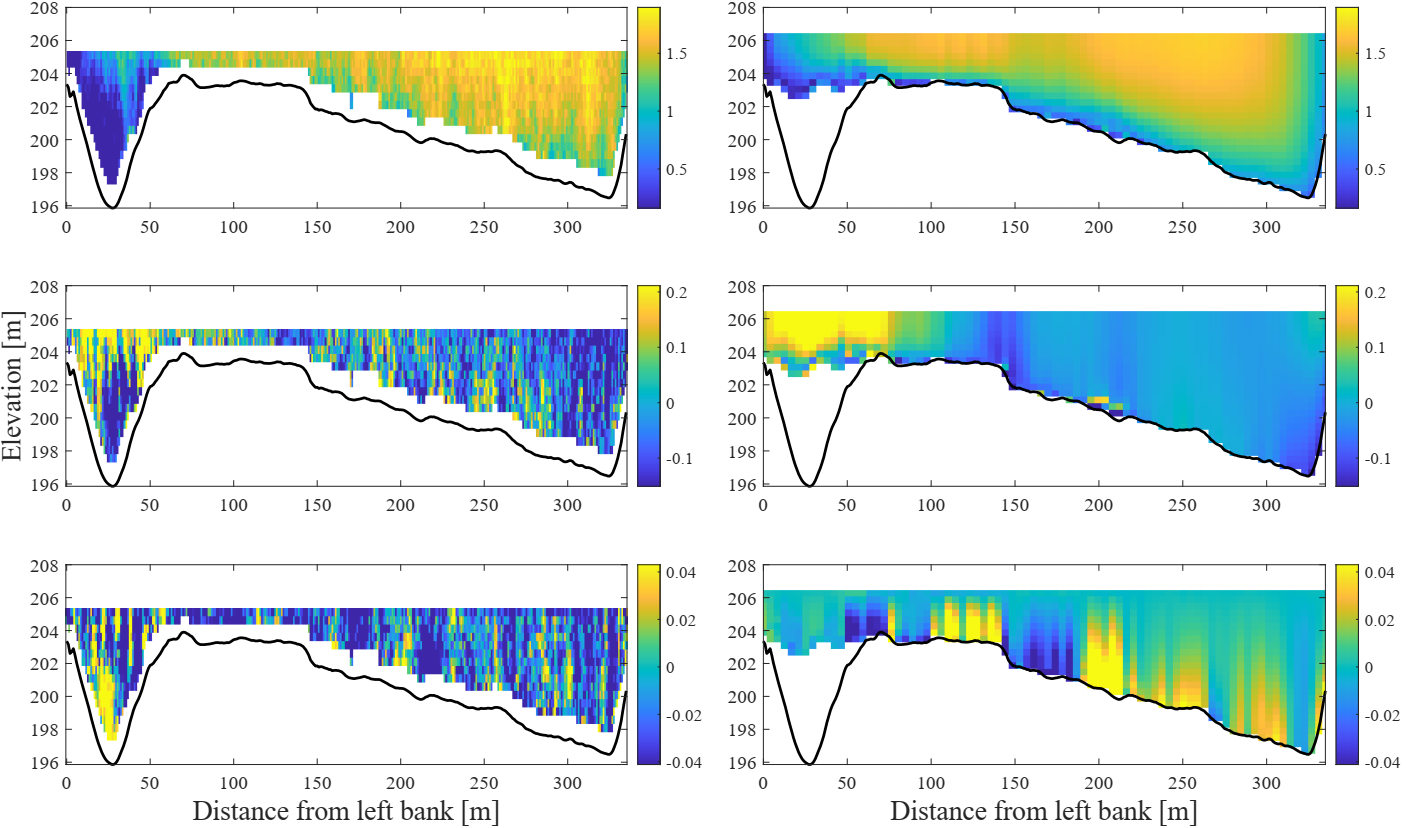


Figure S1. Continued.

Q = 3060 m^3^/s, cross section #1
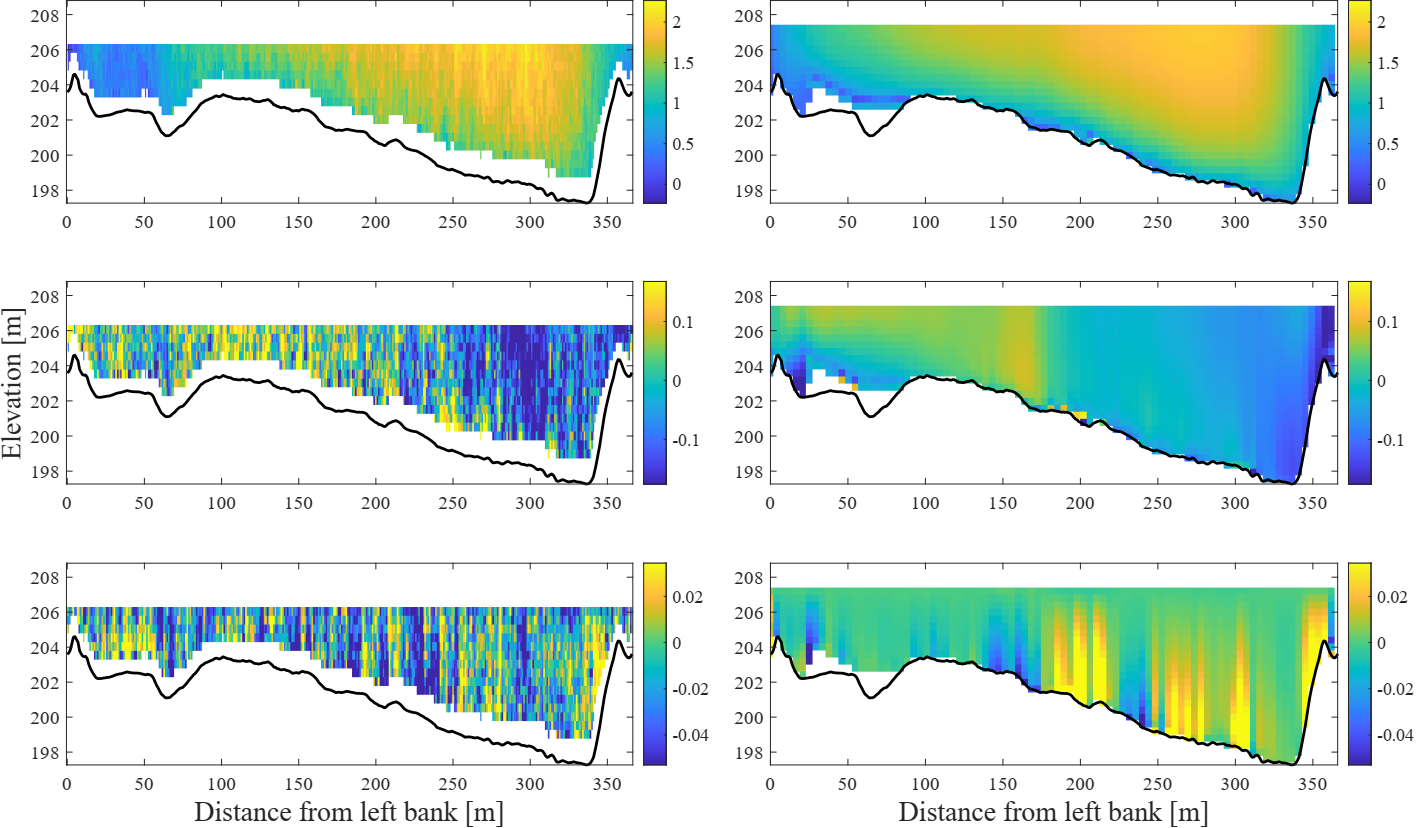


Q = 3060 m^3^/s, cross section #2
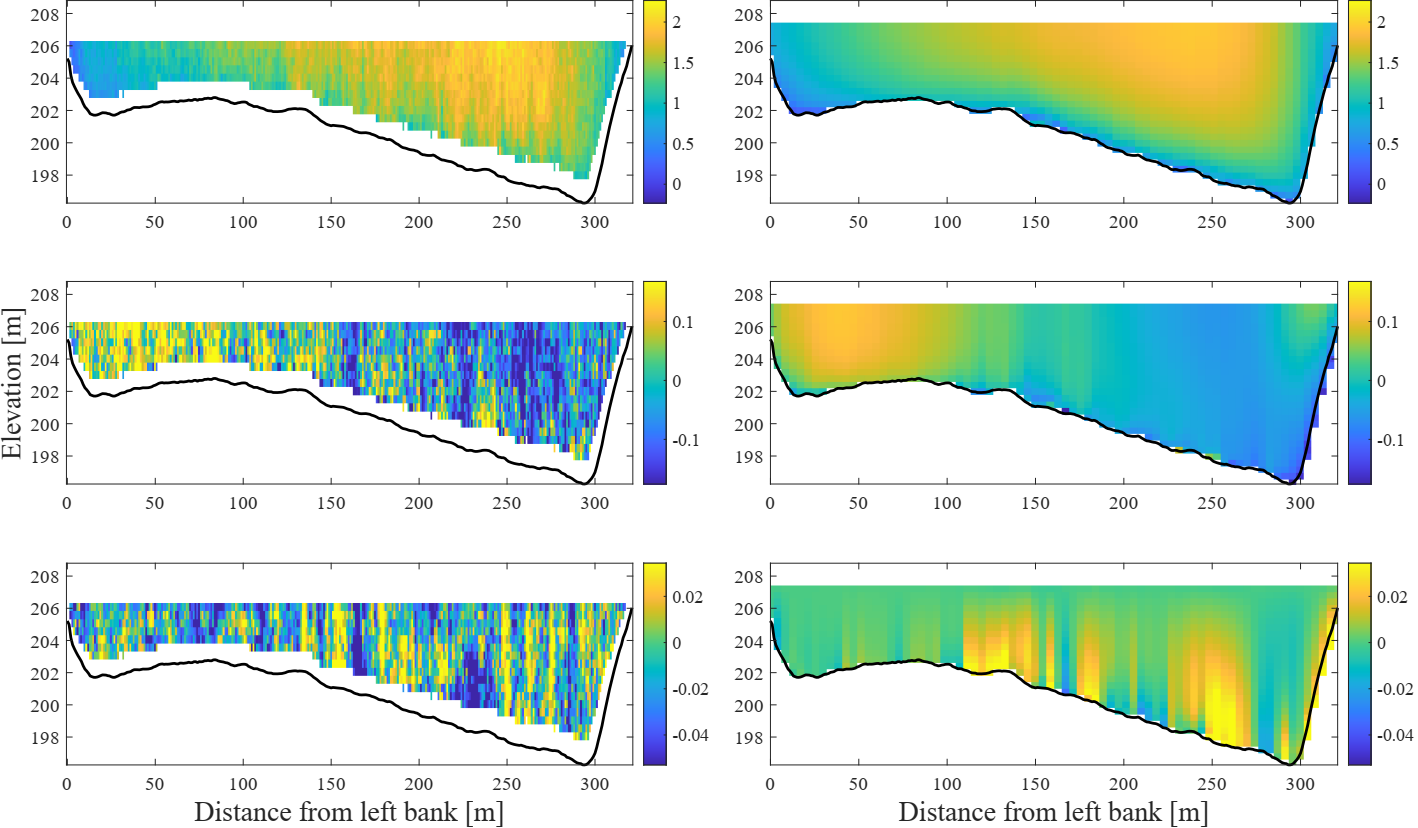


Figure S1. Continued.

Q = 3060 m^3^/s, cross section #3
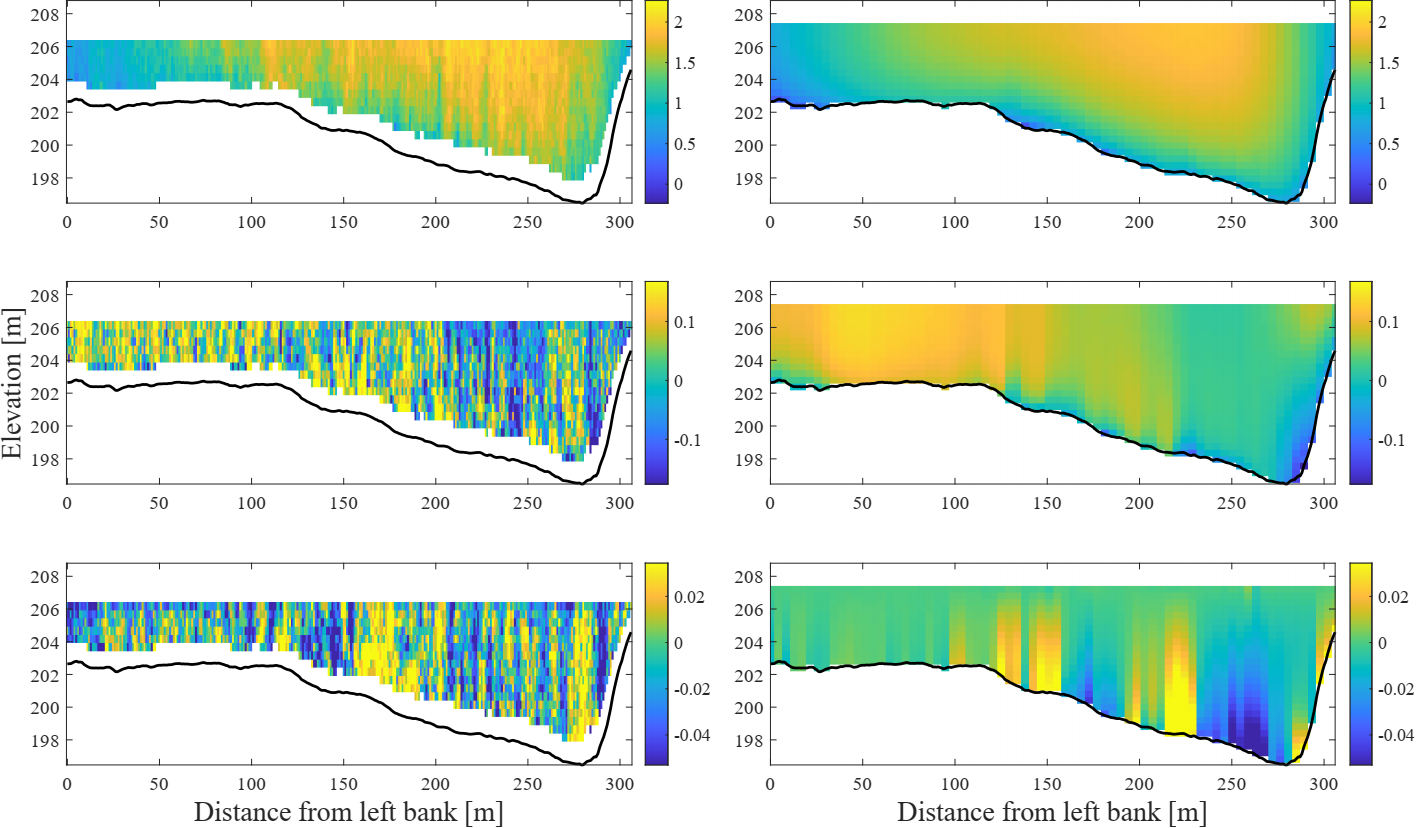


Q = 3060 m^3^/s, cross section #4
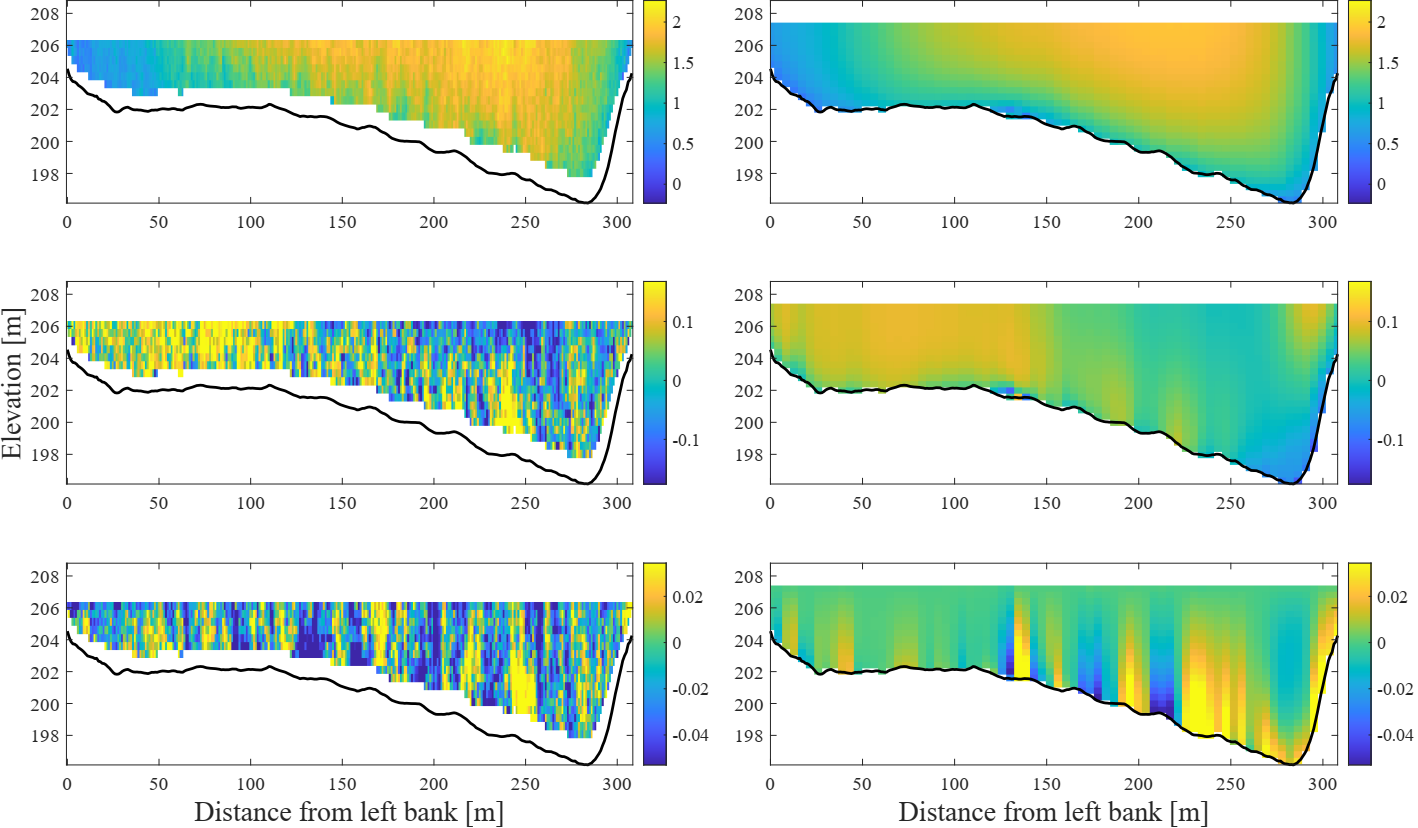


Figure S1. Continued.

Q = 3060 m^3^/s, cross section #5
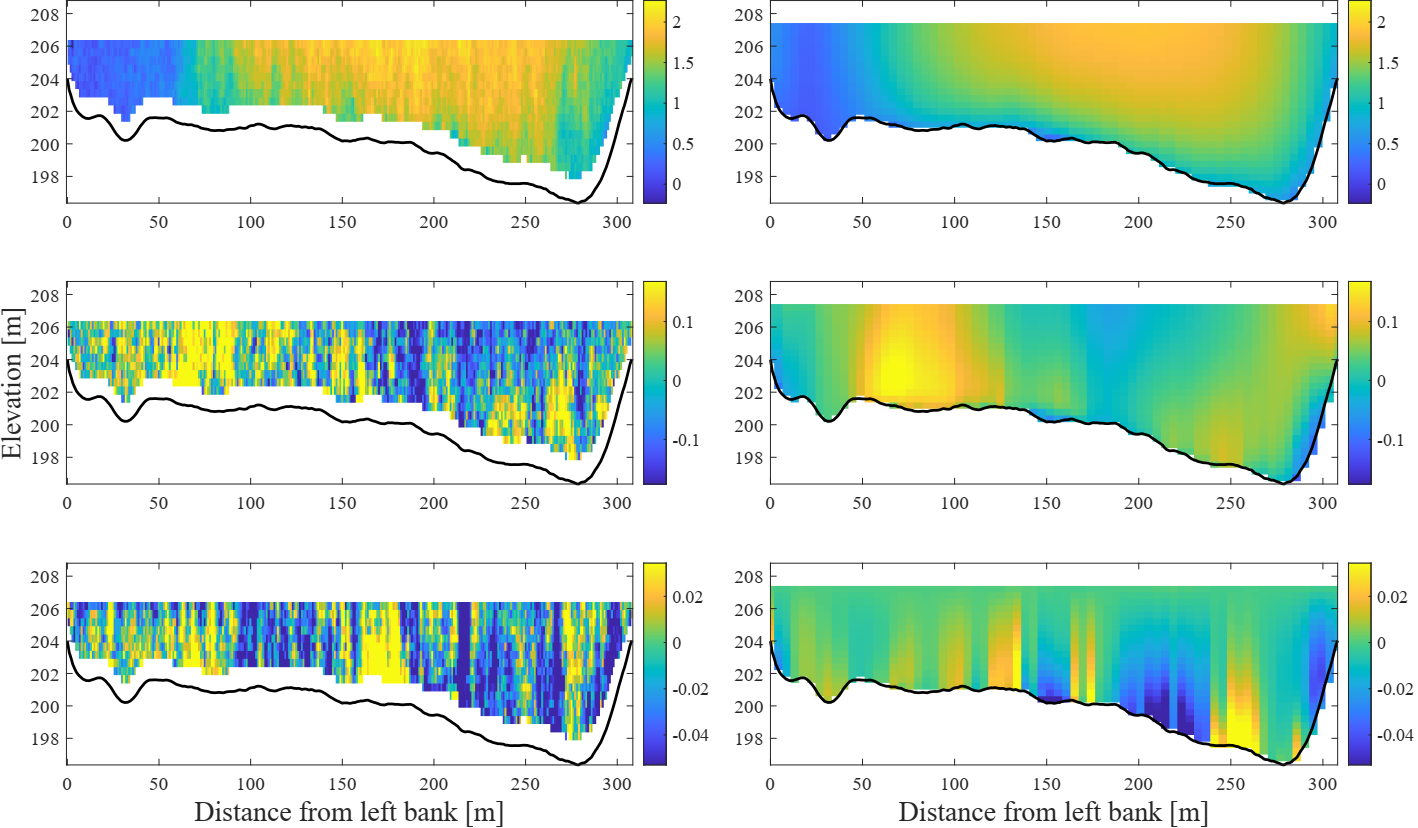


Q = 3060 m^3^/s, cross section #6
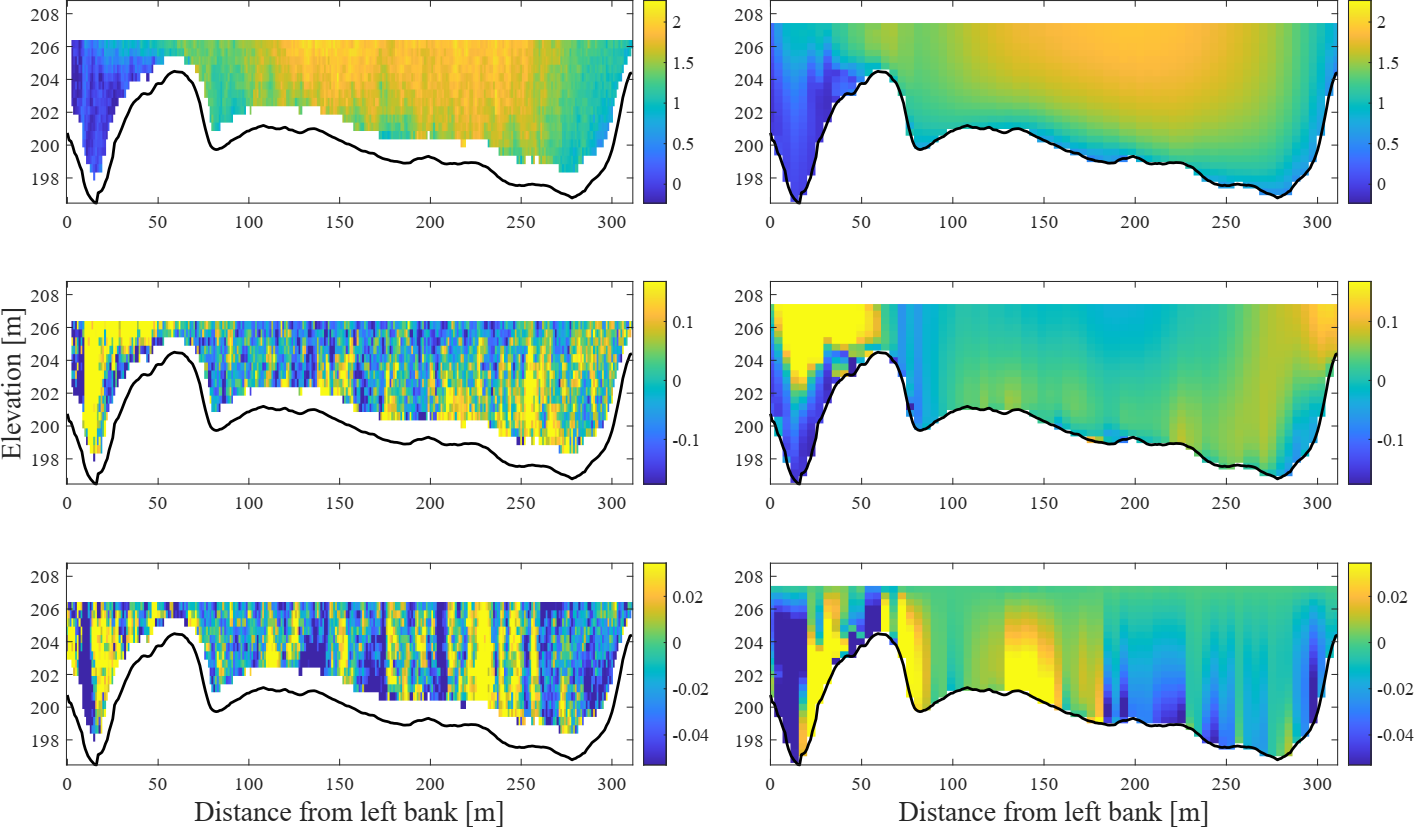


Figure S1. Continued.

Q = 3060 m^3^/s, cross section #7
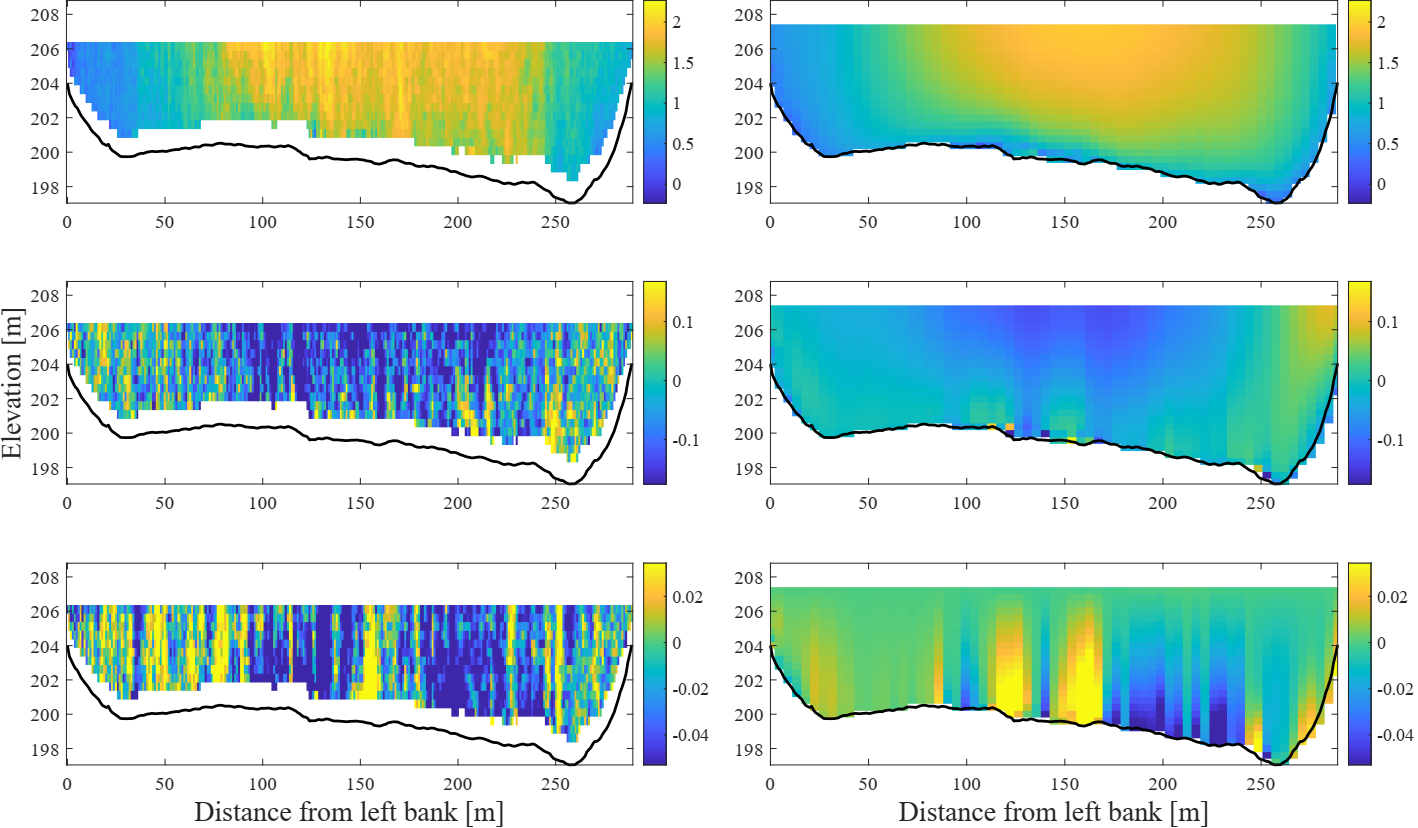


Q = 3060 m^3^/s, cross section #8
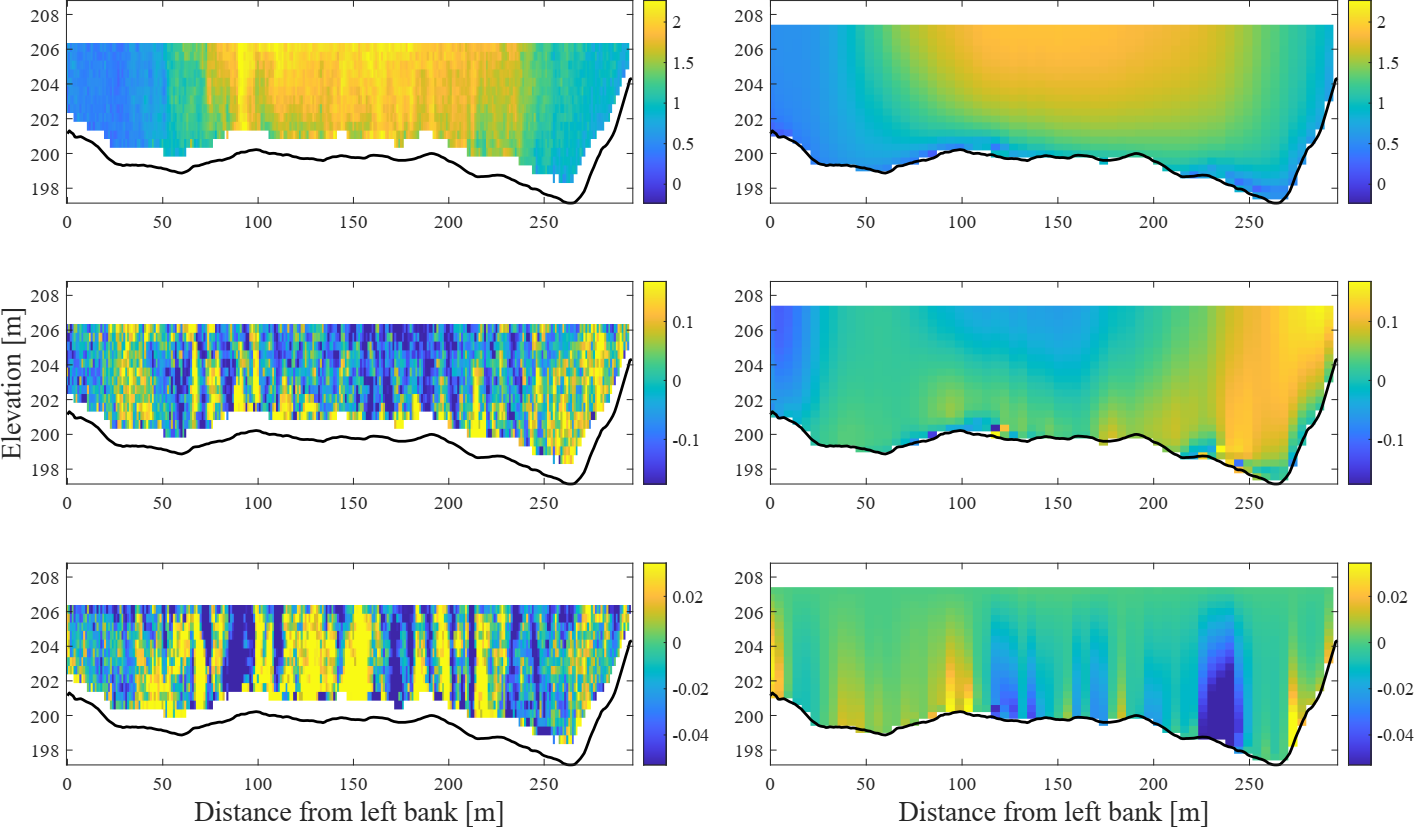


Figure S1. Continued.

Q = 3060 m^3^/s, cross section #9
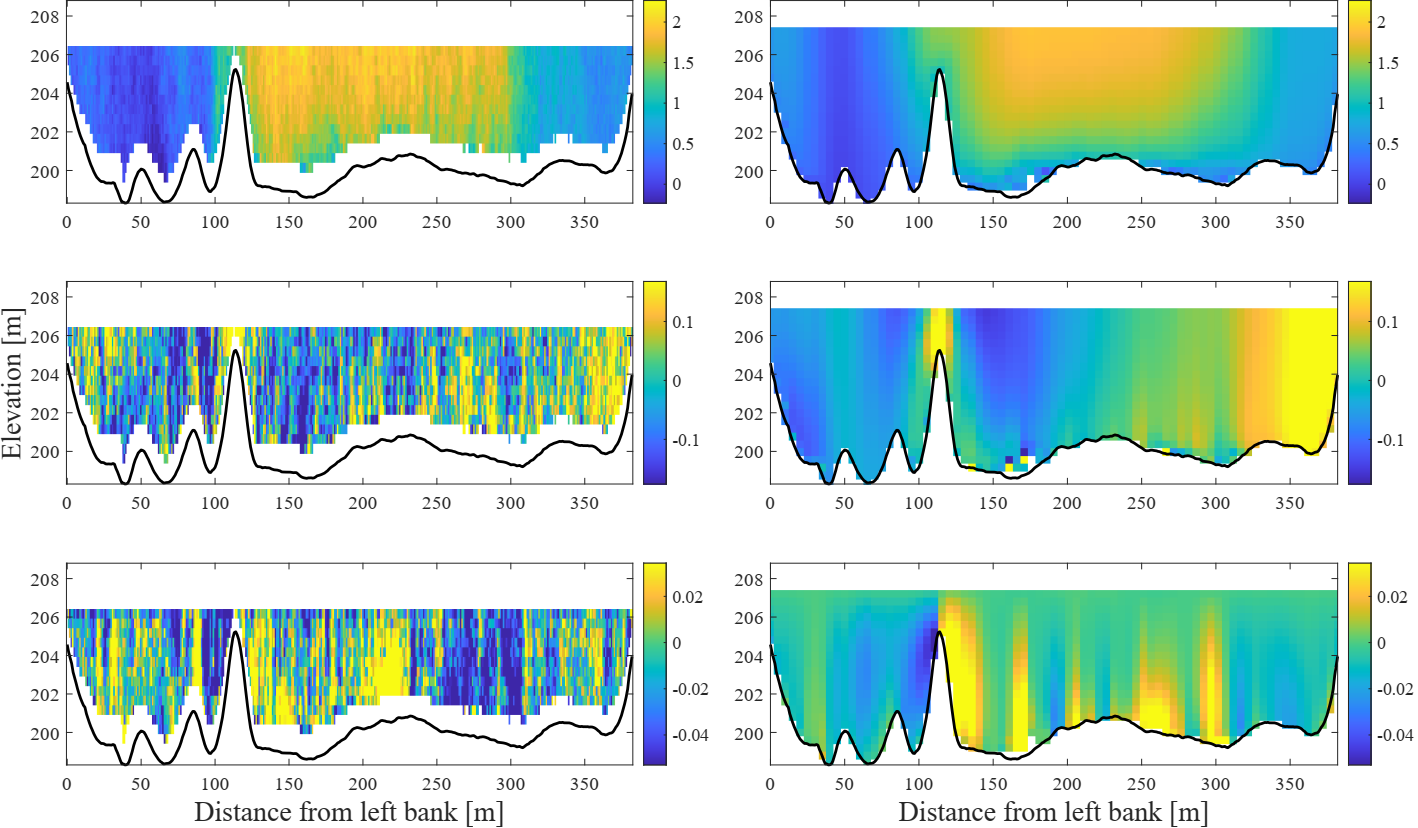


Q = 3060 m^3^/s, cross section #10
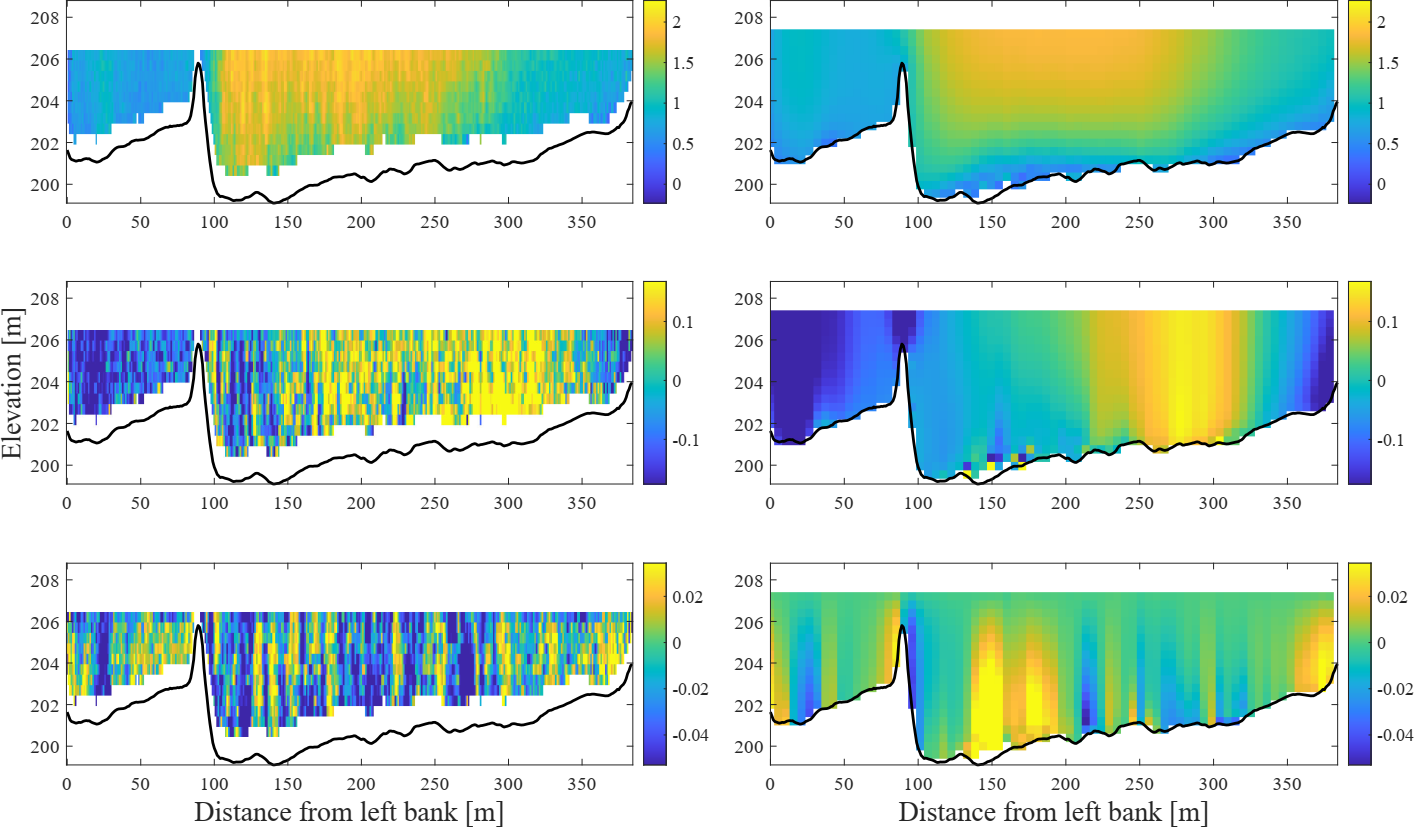


Figure S1. Continued.

| Q = 2282 m^3^/s, cross section #1 | Q = 2282 m^3^/s, cross section #2 |
| --- | --- |
| 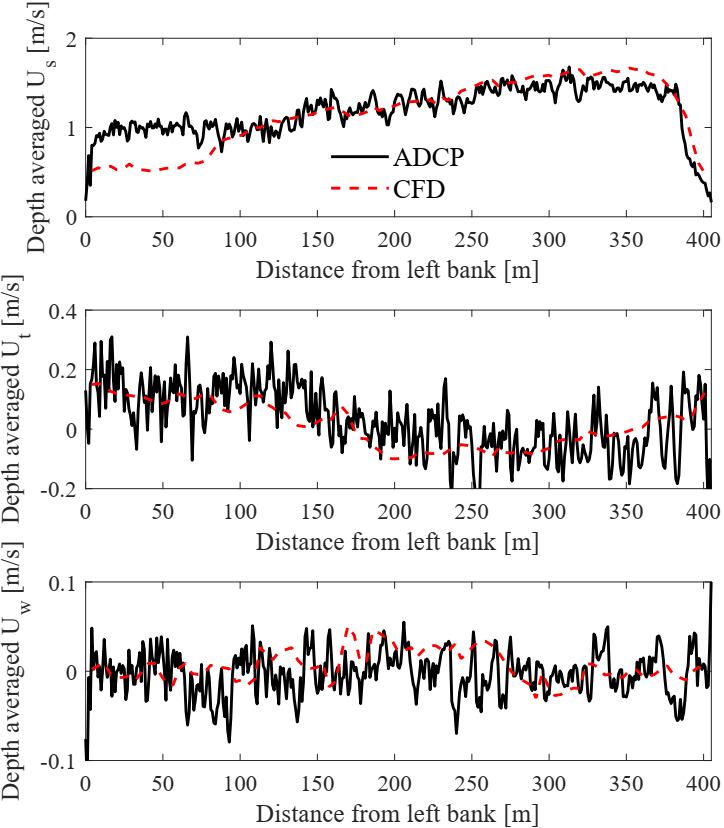 | 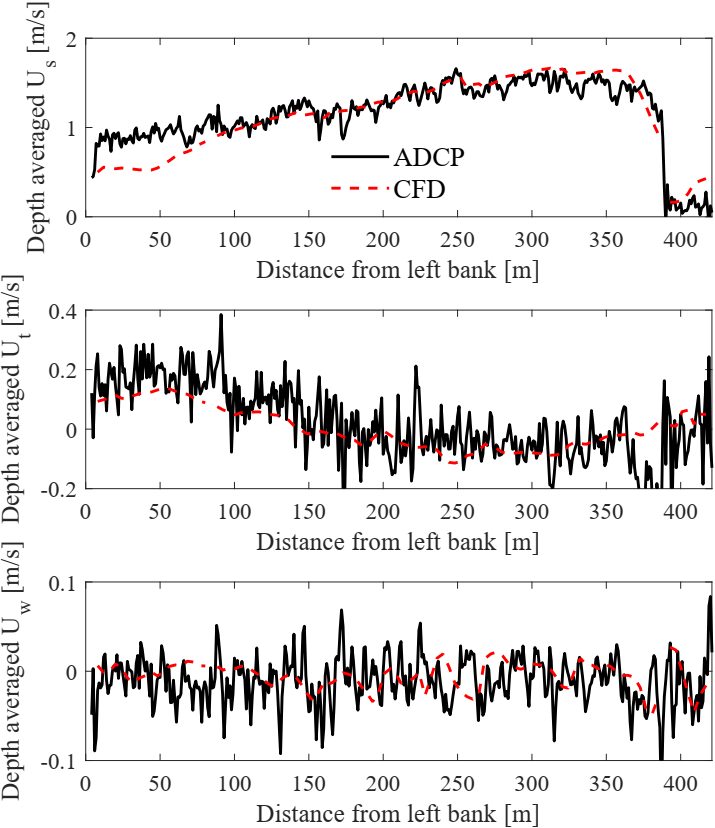 |
| Q = 2282 m^3^/s, cross section #3 | Q = 2282 m^3^/s, cross section #4 |
| 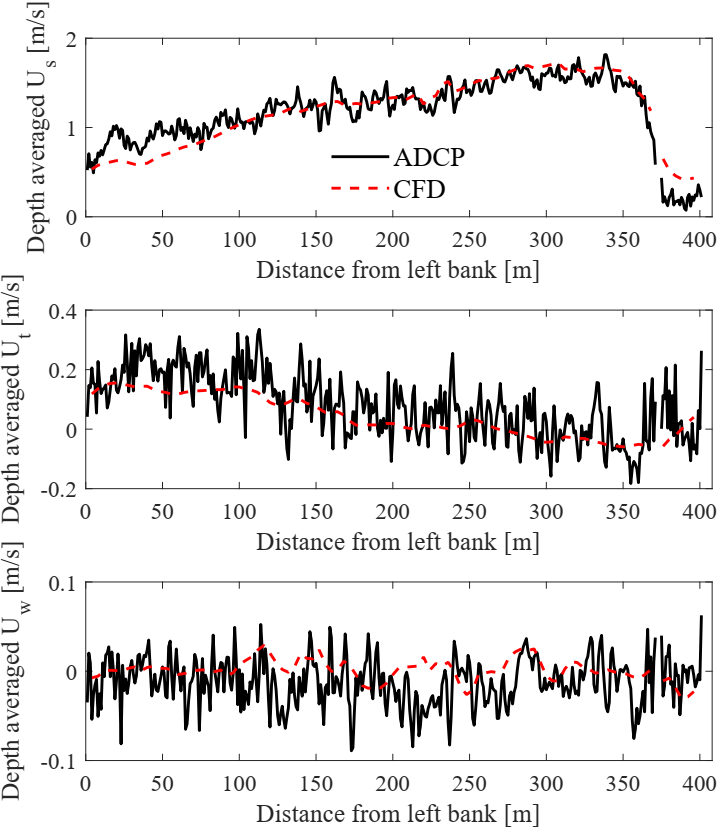 | 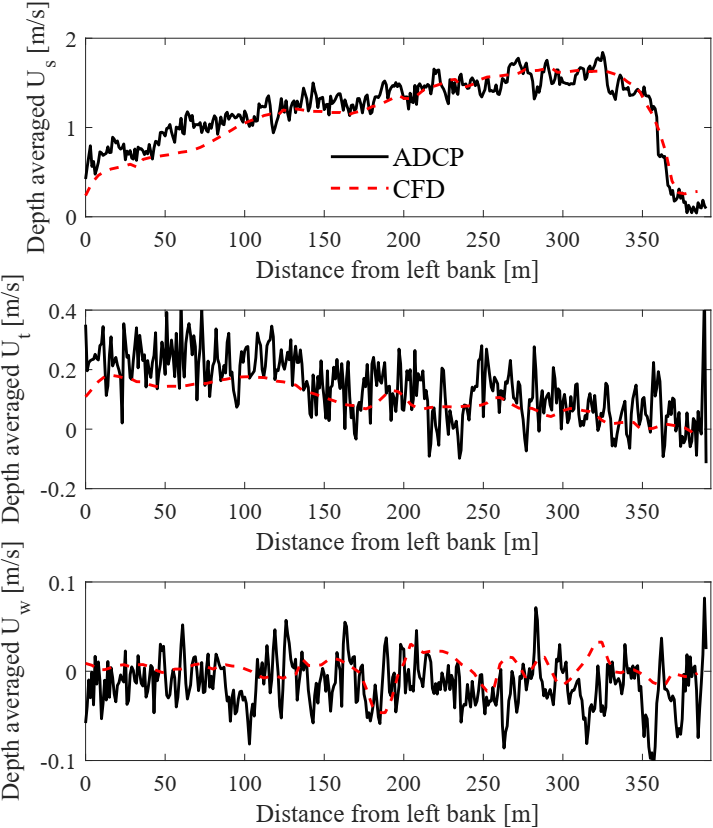 |
|  |  |

Figure S2. Comparison between computational fluid dynamics (CFD) model and acoustic Doppler velocity profiler (ADCP) measurement for depth averaged velocities (Us: streamwise velocity; Ut: transverse velocity, Uw: vertical velocity).

| Q = 2282 m^3^/s, cross section #5 | Q = 2282 m^3^/s, cross section #6 |
| --- | --- |
| 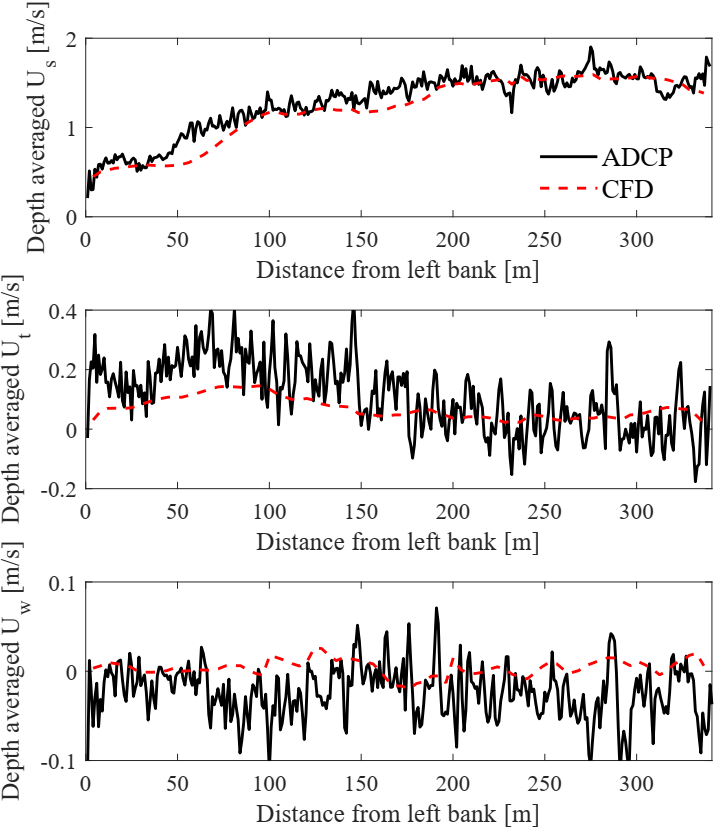 | 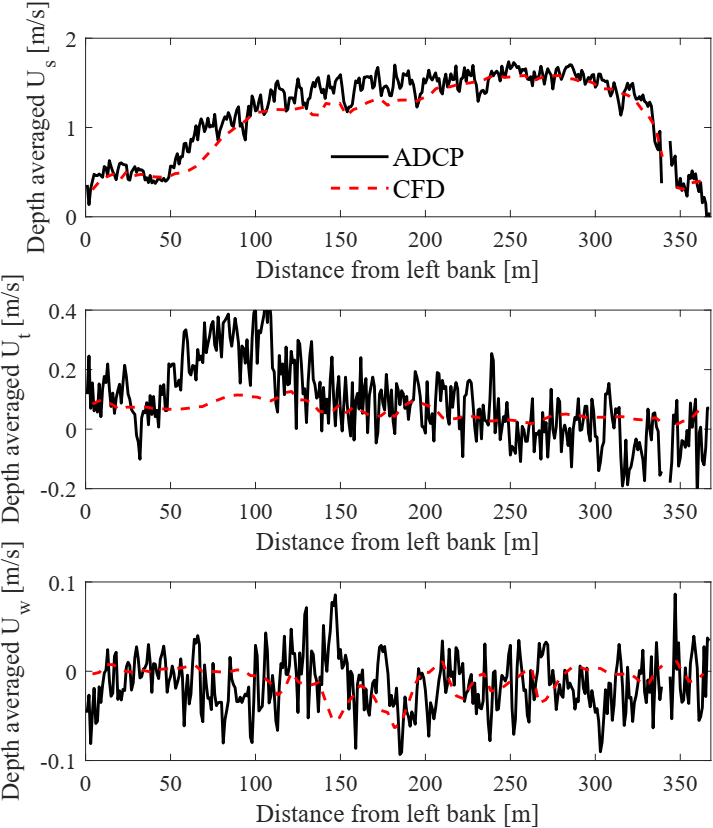 |
| Q = 2282 m^3^/s, cross section #7 | Q = 2282 m^3^/s, cross section #8 |
| 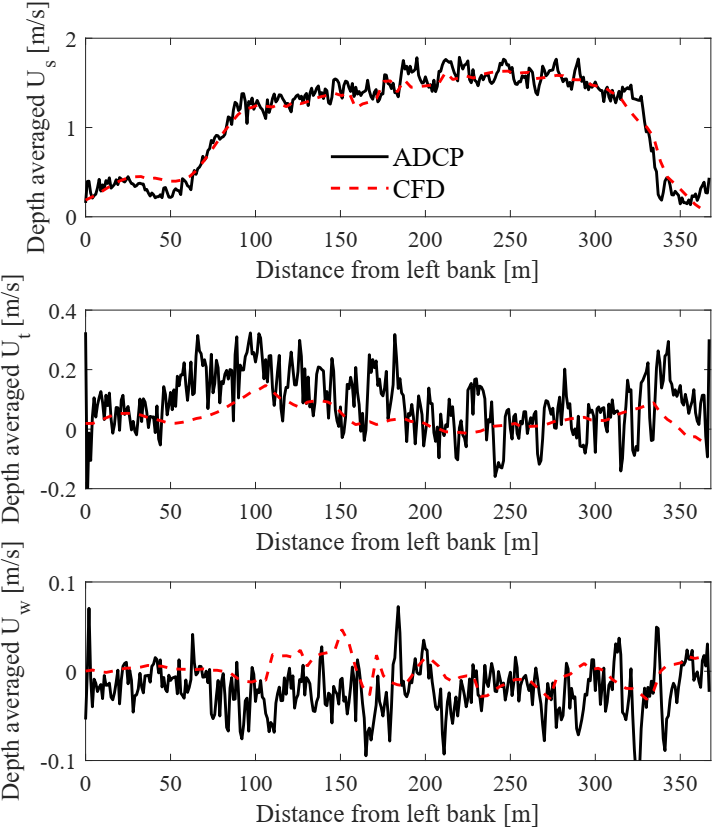 | 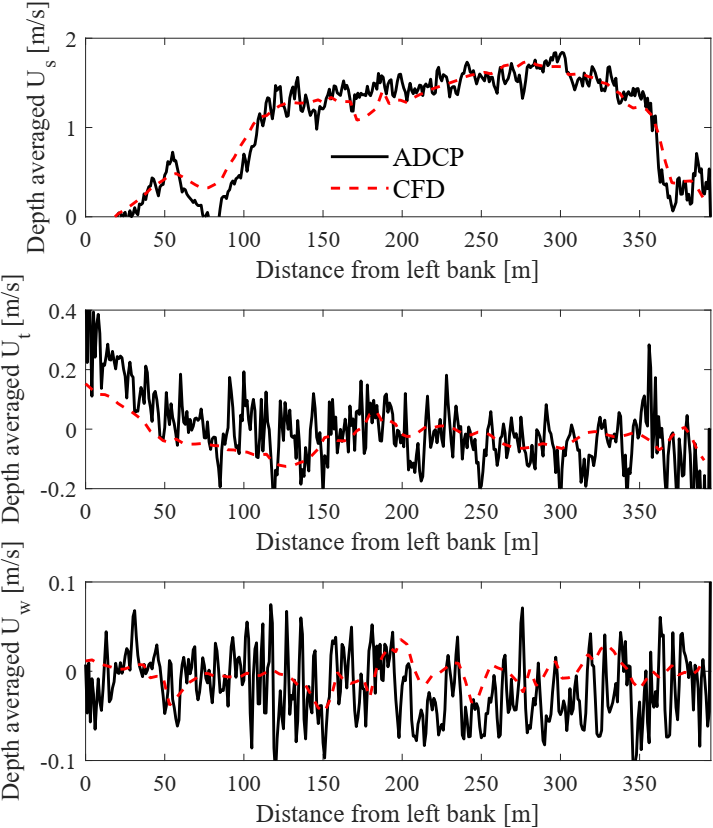 |
|  |  |

Figure S2. Continued.

| Q = 2282 m^3^/s, cross section #9 | Q = 2282 m^3^/s, cross section #10 |
| --- | --- |
| 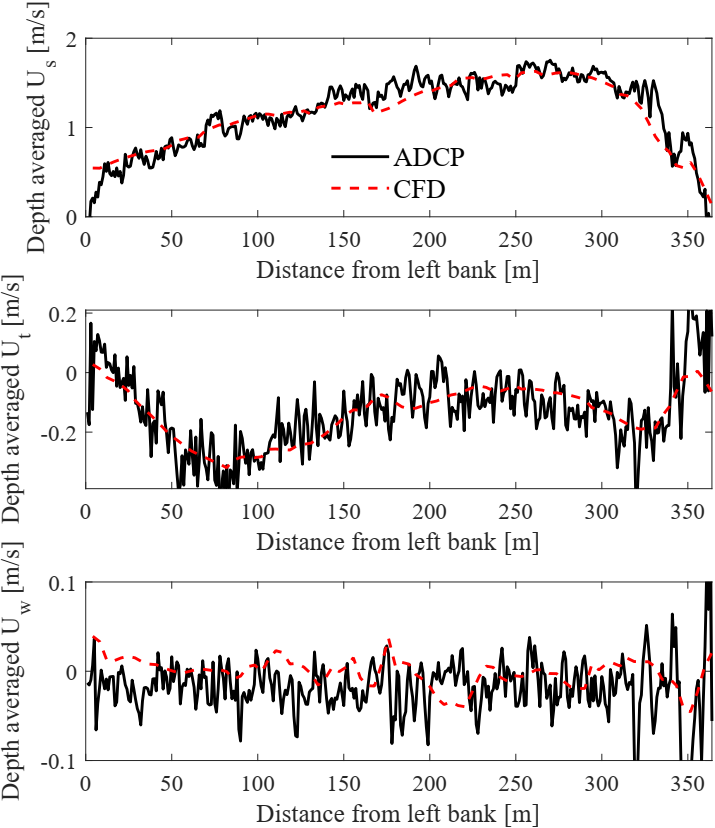 | 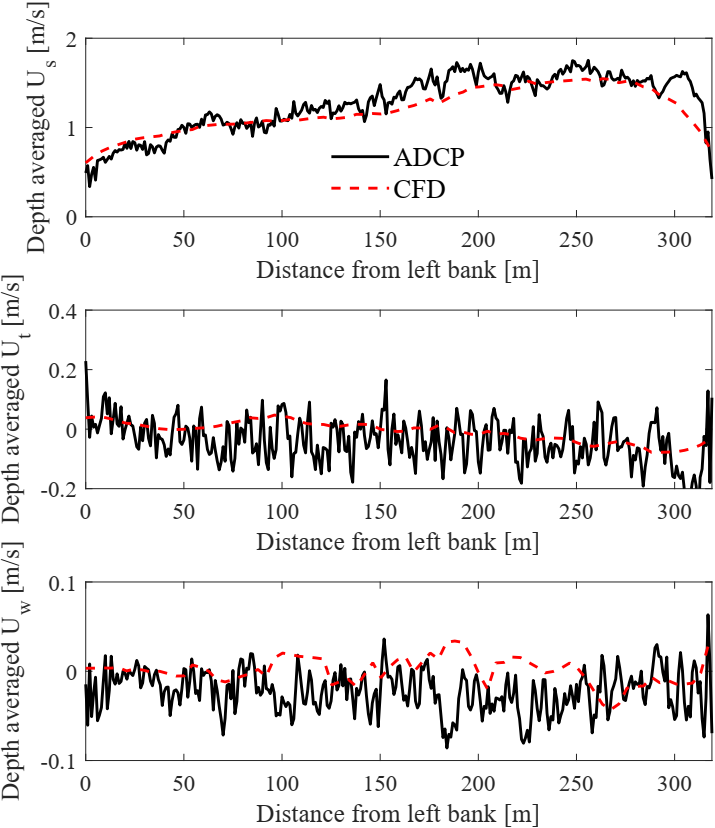 |
| Q = 2282 m^3^/s, cross section #11 | Q = 2282 m^3^/s, cross section #12 |
| 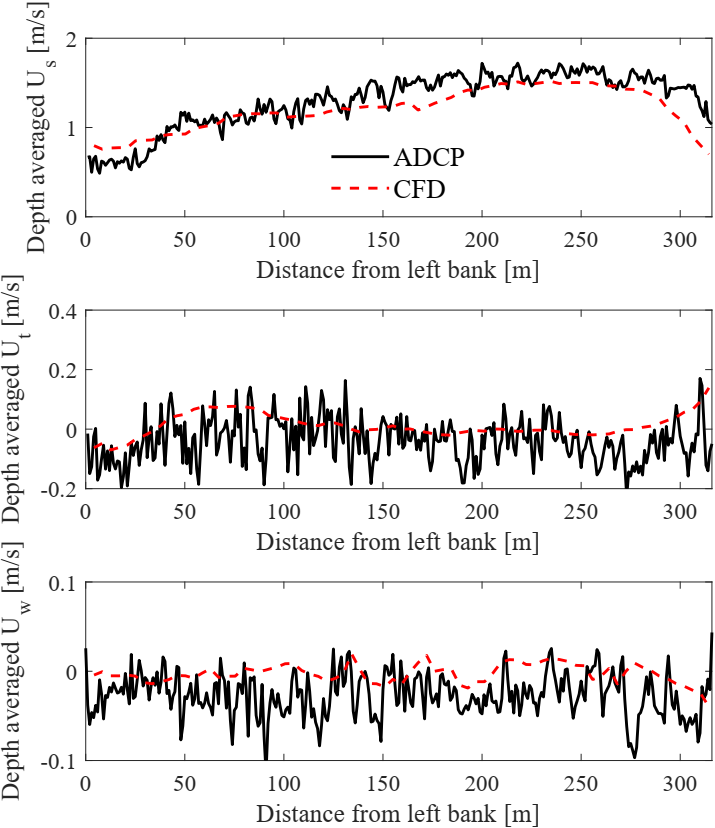 | 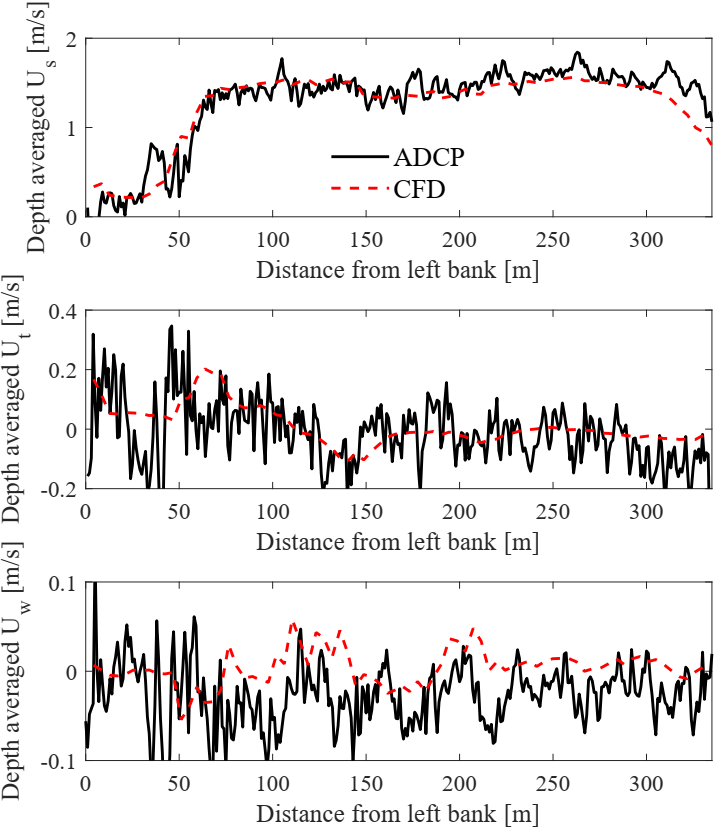 |
|  |  |

Figure S2. Continued.

| Q = 3060 m^3^/s, cross section #1 | Q = 3060 m^3^/s, cross section #2 |
| --- | --- |
| 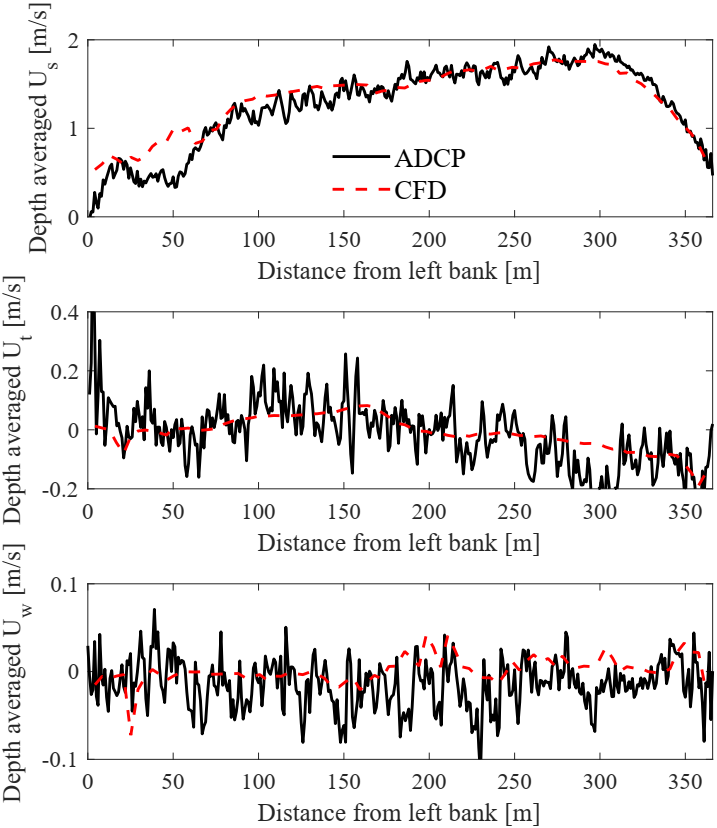 | 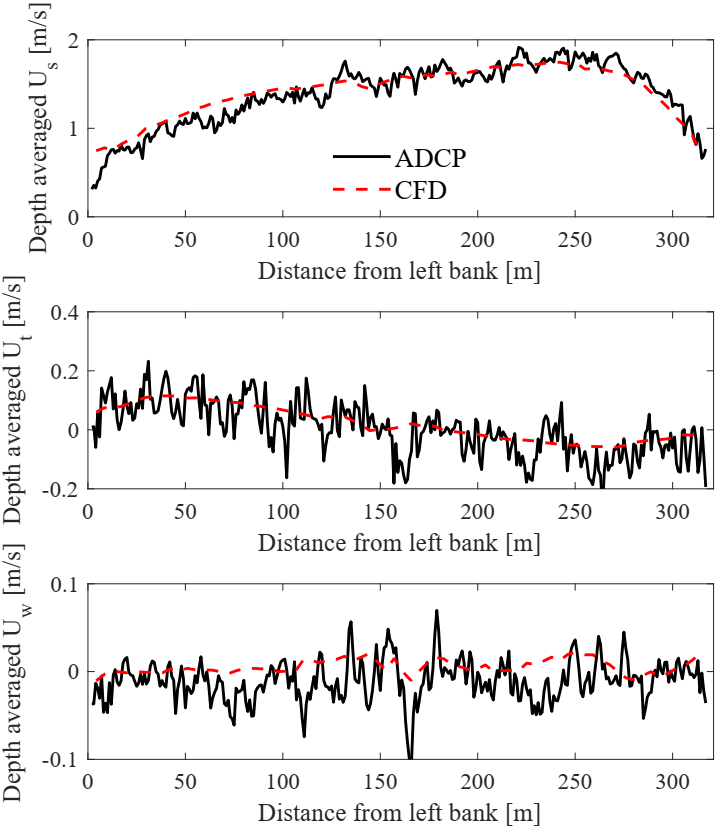 |
| Q = 3060 m^3^/s, cross section #3 | Q = 3060 m^3^/s, cross section #4 |
| 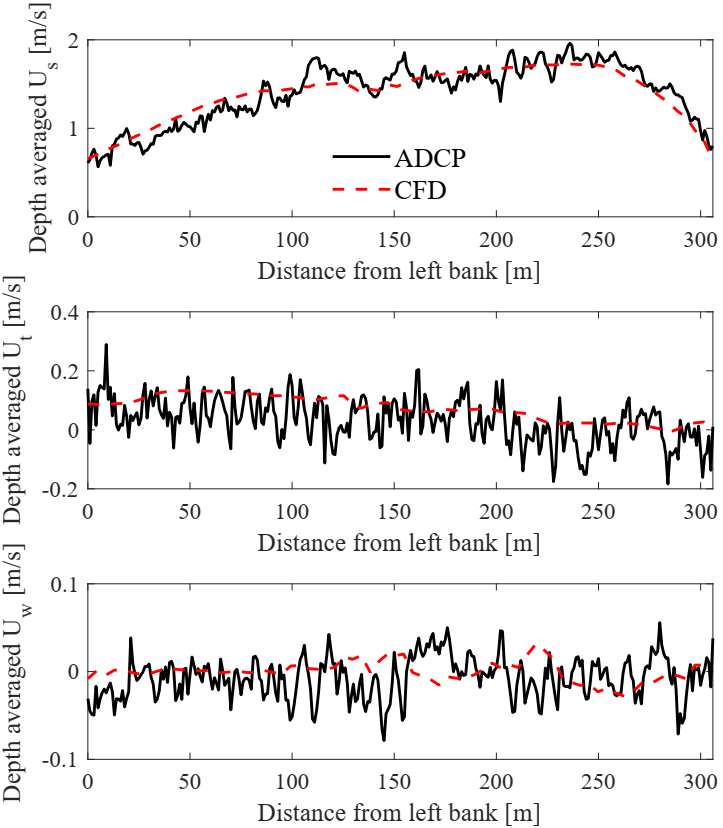 | 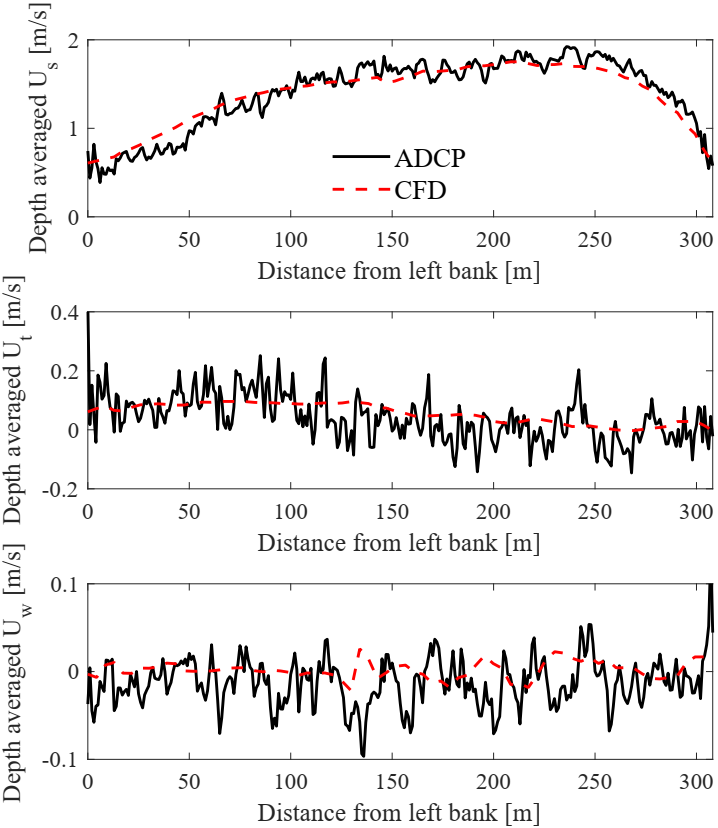 |
|  |  |

Figure S2. Continued.

| Q = 3060 m^3^/s, cross section #5 | Q = 3060 m^3^/s, cross section #6 |
| --- | --- |
| 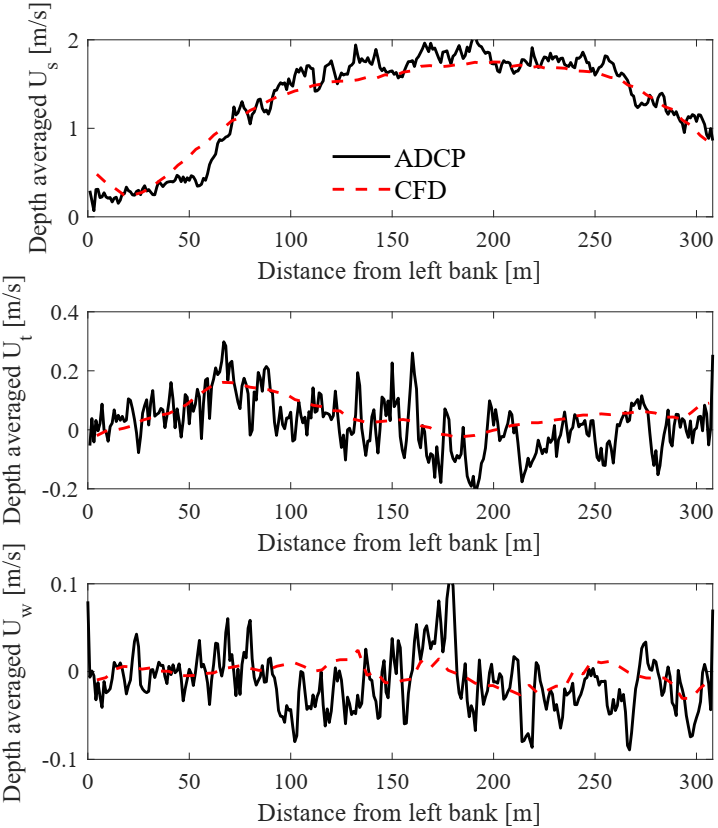 | 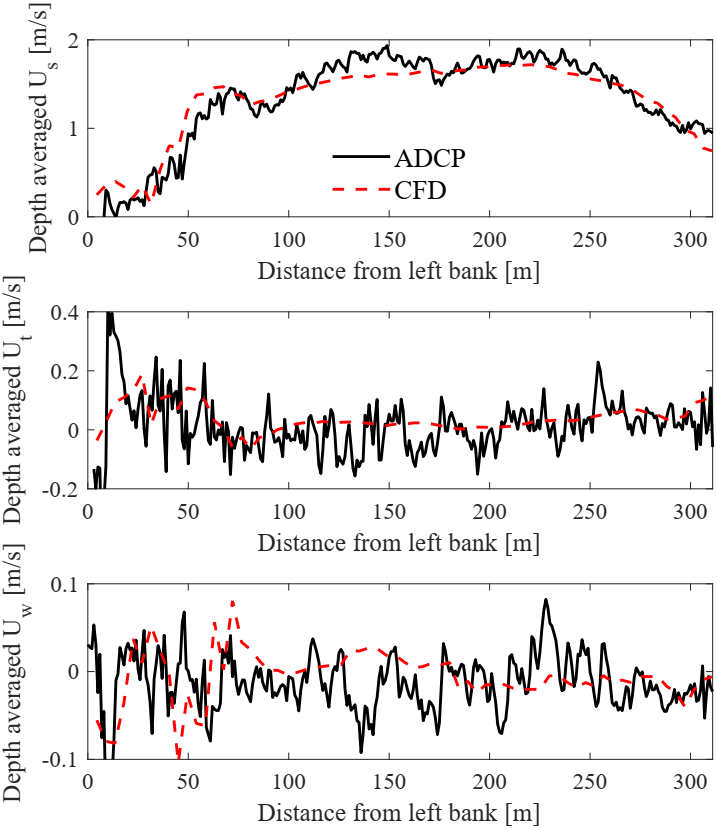 |
| Q = 3060 m^3^/s, cross section #7 | Q = 3060 m^3^/s, cross section #8 |
| 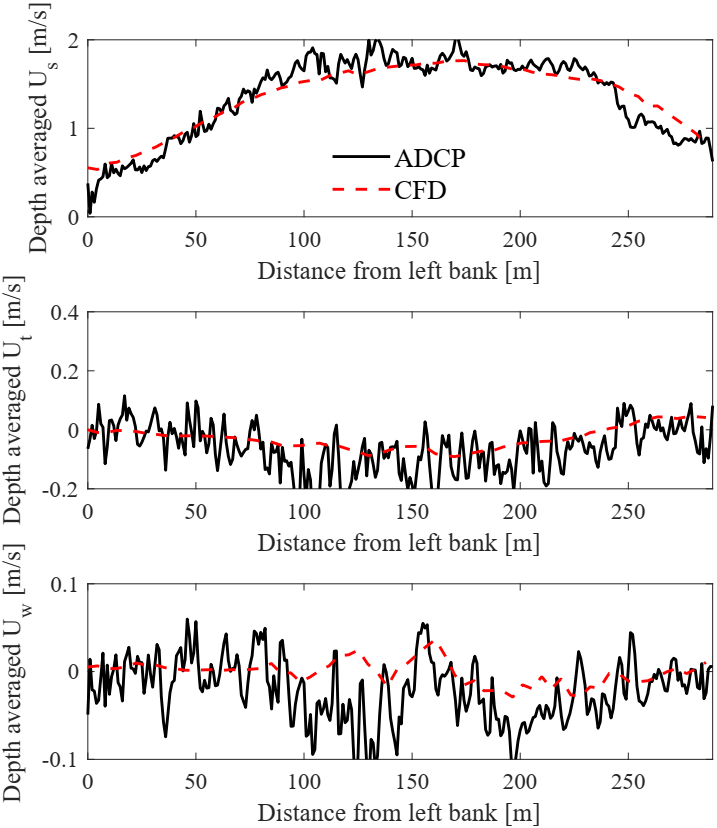 | 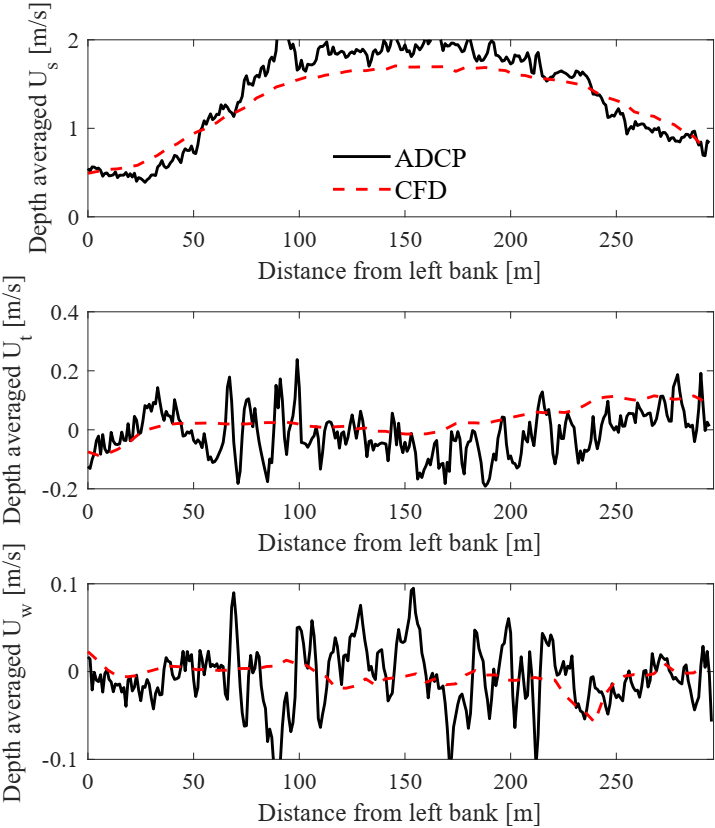 |
|  |  |

Figure S2. Continued.

| Q = 3060 m^3^/s, cross section #9 | Q = 3060 m^3^/s, cross section #10 |
| --- | --- |
| 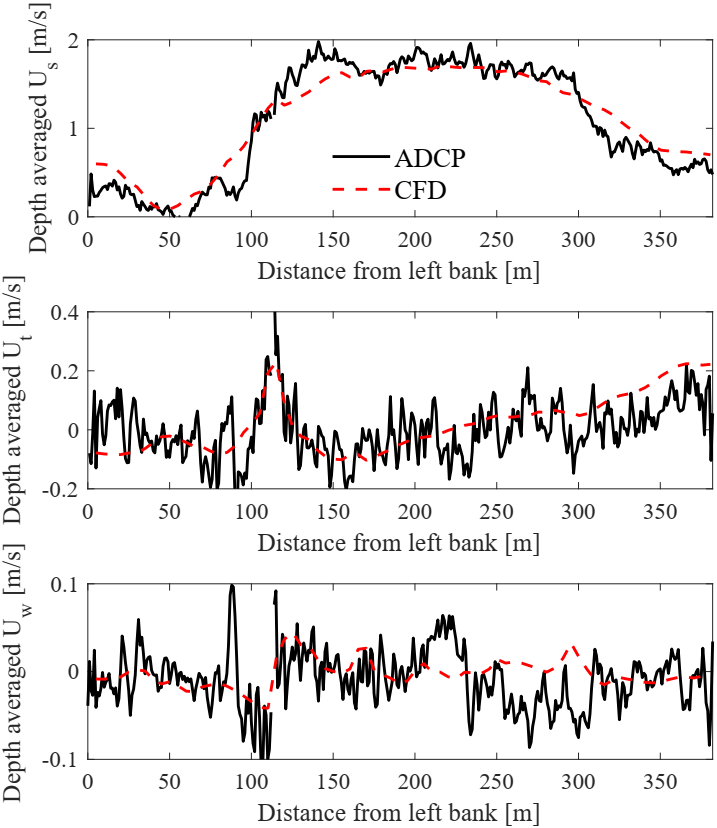 | 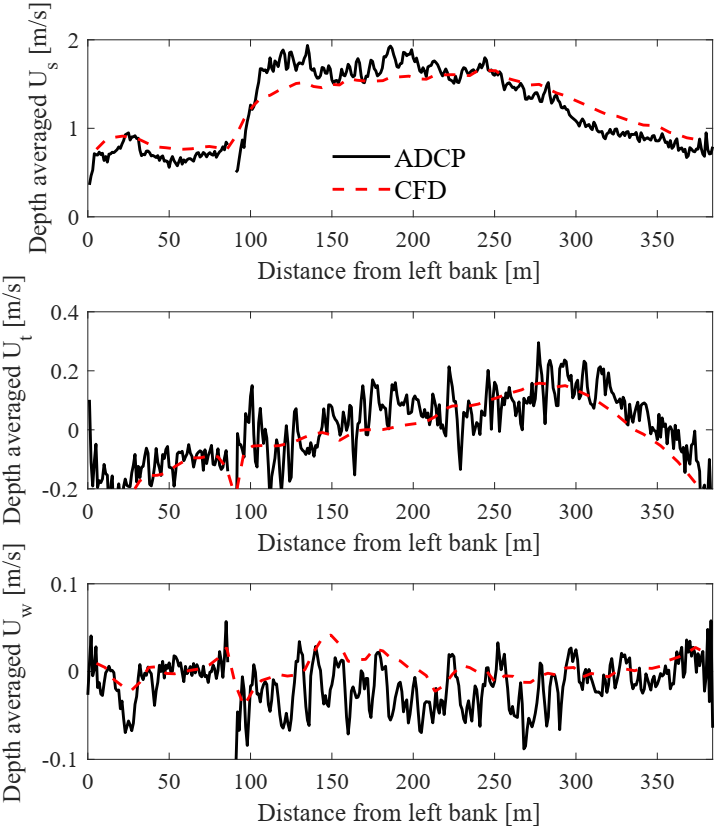 |
|  |  |
|  |  |
|  |  |

Figure S2. Continued.

Figure S3. Predicted egg settling locations using the criterion of shear velocity. Color bar presents river-bed elevation in meters. River miles are indicated in the plot by red triangles.

Figure S4. Predicted egg settling locations using the criterion of vertical turbulence intensity. Color bar presents river-bed elevation in meters. River miles are indicated in the plot by red triangles.

Figure S5. Vertical distribution of hydrodynamic-inferred egg settling locations using the criterion of shear velocity. (a) Number of counts as a function of different heights (z) above the riverbed; (b) number of counts as a function of the normalized heights which are normalized using flow depth (H); (c) probability distribution function (PDF) of the occurrence as a function of z; (d) PDF of the occurrence as a function of z/H.

Figure S6. Vertical distribution of hydrodynamic-inferred egg settling locations using the criterion of vertical turbulence intensity. (a) Number of counts as a function of different heights (z) above the riverbed; (b) number of counts as a function of the normalized heights which are normalized using flow depth (H); (c) probability distribution function (PDF) of the occurrence as a function of z; (d) PDF of the occurrence as a function of z/H.
